# Comparison of behavioural tendencies between “dangerous dogs” and other domestic dog breeds – Evolutionary context and practical implications

**DOI:** 10.1111/eva.13479

**Published:** 2022-10-19

**Authors:** Alexa Hammond, Thomas Rowland, Daniel S. Mills, Małgorzata Pilot

**Affiliations:** ^1^ School of Life Sciences University of Lincoln Lincoln UK; ^2^ Museum and Institute of Zoology Polish Academy of Sciences Gdańsk Poland; ^3^ Faculty of Biology University of Gdańsk Gdańsk Poland

**Keywords:** aggressive behaviour, breed‐specific legislation, domestic dog, evolution of behavioural traits, heritability, impulsivity

## Abstract

Aggressive behaviour by dogs is a considerable social problem, but the ability to predict which individuals may have increased aggressive tendencies is very limited, restricting the development of efficient preventive measures. There is a common perception that certain breeds are more likely to exhibit aggressive behaviour, which has contributed to the introduction of breed‐specific legislation. The rationale for such legislation explicitly assumes high heritability of this trait while also implying relatively little variation within breeds; these assumptions are largely untested. We compared behavioural tendencies between 8 breeds that are subject to legislation in at least one country and 17 breeds that are not subject to legislation using two validated psychometric tools: the Dog Impulsivity Assessment Scale (DIAS), which scores elements of impulsivity, including a tendency for aggressive behaviour, and Positive and Negative Activation Scale (PANAS), which scores sensitivity to positive and negative stimuli (which may trigger aggressive responses). We found that the two groups of breeds do not differ significantly in the specific DIAS factor relating to aggressive behaviour, “Aggression Threshold and Response to Novelty”, or any other DIAS and PANAS factors. We found large variations in all behavioural tendencies measured by both psychometric scales within both groups and within each breed studied. Taken together, our findings indicate that breed alone is not a reliable predictor of individual behavioural tendencies, including those related to aggression, and therefore breed‐specific legislation is unlikely to be an effective instrument for reducing risk.

## 1 INTRODUCTION

Aggressive behaviour in animals is shown in a wide range of contexts. Animals can display conspecific and heterospecific aggression in relation to competition for resources (such as food and space) and offspring protection, while conspecific aggression can also occur in relation to competition for mates and the maintenance of hierarchies in groups (Peiman & Robinson, 2010). The evolution of aggressive behaviour, and its genetic and neurobiological bases, are all of theoretical interest within the broader context of the evolution of animal behavioural traits (De Boer et al., 2003; Dubois & Giraldeau, 2005; Maynard‐Smith & Price, 1973; Nelson & Trainor, 2007), but also of practical importance in the context of both human aggression towards conspecifics as well as interactions between humans and domesticated animals.

The domestic dog (*Canis lupus familiaris*), the only large carnivore to be domesticated, can pose a significant danger to humans from aggressive encounters. Aggressive behaviour may not only occur in the context of agonistic (offensive‐defensive) encounters, but also as a result of predatory responses, or simply by accident (Mills, 2017). Dog bites may result in serious injuries or death, and are a major public health problem in some countries (Polo et al., 2015). Predatory behaviour towards humans is considered to be an uncommon cause of dog bites, even fatal ones (Van Der Voorded & Rijken, 2017). While accidental bites (e.g. during play) and redirected bites (e.g. in situations when dog owners try to prevent intraspecific aggression) may be common, their accidental nature requires different strategies for their prevention. Therefore, it is understandable that there is the greatest legal focus on the risk from agonistic encounters, in which bites are typically the end point of an escalating series of threats (Siniscalchi et al., 2018). Domestication is thought to have involved selection for reduced agonistic aggression towards humans (Hare & Tomasello, 2005). However, such a trait cannot be completely eliminated from dog populations, because it remains a vital defence mechanism, and some dogs are bred for guarding and protection work, where elements of agonistic display may be desirable. Therefore, considerable variation in the typical threshold for the expression of aggressive responses in particular contexts can be expected between breeds (Mehrkam & Wynne, 2014).

Certain dog breeds – predominantly those historically used for guarding work or dog fighting – are commonly considered to be more likely to exhibit problematic aggressive behaviour, and legislation aimed at preventing dog attacks on humans is typically focused on restricting or prohibiting ownership of such breeds (Allcock & Campbell, 2021; Creedon & Ó'Súilleabháin, 2017; Súilleabháin, 2015). For example, the Dangerous Dogs Act (1991) in the UK prohibits the ownership of four breed types, the Fila Brasileiro, Dogo Argentino, Japanese Tosa and the Pit Bull Terrier. An explicit assumption underpinning the rationale for breed‐specific legislation (BSL) is a high heritability of the tendency towards aggression in dogs, with an implicit assumption that there is relatively little variation within breeds. High heritability is necessary for a trait to become breed‐specific or breed‐typical, as occurs with breed‐related morphological traits. In theory, breed‐typical behavioural traits could be achieved also by specific training provided to all individuals representing the breed, but in such case, any legislative solution should address the training rather than the breed. Large variation of a heritable trait within breeds implies that such trait is not breed‐specific; if the breed is not a good predictor of the behaviour of an individual, this undermines the rationale for breed‐specific legislation.

Heritability of aggressive behaviour in canids has been demonstrated through the selective breeding of foxes (*Vulpes vulpes*), where aggressive and tame strains were created via assortative breeding of individuals on the basis of their response towards humans (Kukekova et al., 2008). These strains show differentiation in DNA sequences of genes involved in key pathways regulating neurological processing as well as expression levels of these genes in the brain (Wang et al., 2018). Stranger‐directed aggression has been shown to be among behavioural traits with the highest among‐breed heritability (i.e. behavioural variance across breeds; h^2^ = 0.68) in pure‐bred domestic dogs (MacLean et al., 2019). However, heritability of a trait does not necessarily imply low variation within breeds. A number of behavioural studies have highlighted relatively high variation within breeds compared to variation between breeds in a range of behavioural traits (Duffy et al., 2008; Mehrkam & Wynne, 2014; Svartberg, 2006), including the tendency towards aggression (Fadel et al., 2016). Accordingly, a recent genomic study showed that although most canine behavioural traits have relatively high heritability (h^2^ > 0.25), breed explained only 9% of behavioural variation among individuals (Morrill et al., 2022). This may suggest that variation in the tendency towards aggression has been maintained in breeds throughout generations, possibly due to the increased fitness associated with intermediate phenotypes. Alternatively, this variation may result from recent relaxation of selection on behavioural traits in dog breeds, due to a greater focus on morphological over behavioural traits in breed standards.

Breed‐specific legislation has been heavily criticised for its poor scientific basis (Mills & Levine, 2006; Ott et al., 2008) and lack of demonstrable effectiveness, e.g. hospital admissions for dog bite incidents have continued to rise since legislation was introduced (Creedon & Ó'Súilleabháin, 2017). Research focusing specifically on the behavioural differences between breeds affected and not affected by legislation is scarce, but generally does not support BSL. For example, Creedon and Ó'Súilleabháin (2017) found no significant differences between legislated and non‐legislated groups of breeds with regard to either the type of bite inflicted or whether medical intervention was required, indicating no difference in the nature of bites received from the two classes of dog. Other studies have found that behavioural responses of dogs from legislated breeds during a temperament test were classified as appropriate in 95% of individuals tested (Schalke et al., 2008) and did not differ significantly from the behavioural responses of Golden Retrievers (Ott et al., 2008), who are widely considered good family pets. These data could be used to argue that either the legislation provides an incorrect list of breeds which pose an increased bite risk to humans, or that this risk is not breed‐specific.

A wide range of methodologies has been used to compare the behaviour of dog breeds, with the outcome potentially depending on the method used (Mehrkam & Wynne, 2014), which makes it difficult to draw conclusions about specific behavioural differences found between breeds. A number of questionnaires have been developed to evaluate the behaviour of individual dogs over a wide range of contexts (as opposed to the specific context of an experimental setting) (e.g. Hsu & Serpell, 2003; Ley et al., 2009; Mirko et al., 2012). It has been argued that a psychometric approach, which aims to profile biological constructs which underpin behavioural traits (e.g. the executive control of behaviour reflected in the trait “impulsivity”, or sensitivity and responsiveness to aversives commonly reflected in the construct “negative activation”) has the potential to provide a more consistent picture of behavioural tendencies than either arbitrary traits or behavioural experiments (Mills, 2017; Sheppard & Mills, 2002). This approach provides a way to quantify behavioural tendencies across a wide range of contexts. Moreover, it allows rapid data collection from a large number of individuals, which is particularly important when comparing behavioural traits between groups showing large within‐group variation. However, depending on the design, some psychometric questionnaires only provide a tool for assessing individual differences in behaviour, without identifying traits that are meaningful at either a psychological or functional biological level. The phrasing of items is also critical in order to avoid inaccurate answers given either intentionally (e.g. social desirability bias) or resulting from misunderstanding the questions. Therefore, it is very important that the questionnaire is validated if it is to be used as a psychometric tool.

The Dog Impulsivity Assessment Scale (DIAS) (Wright et al., 2011) and the Positive and Negative Activation Scale (PANAS) (Sheppard & Mills, 2002) are two validated instruments of particular relevance for the assessment of behavioural tendencies associated with aggression. Impulsivity is an aspect of executive control that relates to the ability to inhibit behaviour in the presence of salient cues, and thus the ability to control behaviour using a range of cognitive processes. Within the literature on psychiatric disorders involving aggressive behaviour in humans, high impulsivity and poor executive control have been highlighted (Dalley & Robbins, 2017). Previous work on dogs has considered impulsivity in relation to a heightened risk from aggressive behaviour due to reduced preceding threat signals (Amat et al., 2009; Peremans et al., 2003), and this may be very pertinent to the perceived dangerousness of a dog (i.e. ability to predict aggression from lower level threats and take appropriate steps to prevent a bite). The DIAS is composed of three factors (Wright et al., 2011), one of which (“Aggression Threshold and Response to Novelty”) describes a specific behavioural tendency that may be directly relevant to the risk of dog attacks, reflecting a relationship between low aggression threshold and a negative evaluation of novel situations (Wright et al., 2011). Sensitivity to the affective quality of stimuli may be more broadly relevant to the risk of a bite, since individuals that are more aversive to potentially negative stimulus qualities (beyond those associated with the uncertainty of a novel situation), may be expected to become defensive more readily. Moreover, those individuals that are more sensitive to rewards may be more likely to engage in a dispute over resources (Harmon‐Jones et al., 2009) as they might tend to overestimate the value or benefits of a resource. Sensitivity to rewards and aversives can be assessed in dogs using PANAS (Sheppard & Mills, 2002).

The aim of this study was to evaluate some fundamental biological assumptions which underpin breed‐specific legislation by testing whether key traits, as assessed by DIAS and PANAS, show significant differences between a group of breeds that are subject to legislation in at least one country and those that are not, to quantify apparent differences between these groups and examine variability of the traits assessed within each group.

## 2 METHODS

The study was approved under the designated authority of the Research Ethics Committee at the University of Lincoln, UK (UoL2017‐CAB‐001).

### 2.1 Study design

Data were gathered via owner report using an online survey combining demographic information with the Dog Impulsivity Assessment Scale (Wright et al., 2011) and the Positive and Negative Activation Scale (Sheppard & Mills, 2002). The website link was advertised online (via Facebook and relevant dog/breed specific groups, Twitter, pet fora, via the UK Kennel Club including their Bio‐acquisition Research Collaboration page, and the Dog Science Group website). UK breeders of breeds of interest were emailed using the ChampDogs website and encouraged to participate. Participants were informed we were conducting research into breed differences in personality traits, but were not specifically informed about the primary research focus on breed‐specific legislation.

To select legislated dog breeds for inclusion, a list of countries with breed specific legislation was compiled (PETolog, 2016; RSPCA, 2016). Legislated breeds were then selected based on the criterion that the breed is banned or restricted in at least one country that has breed‐specific legislation. Using this criterion, we identified 12 dog breeds and two breed “types” (Dogo Argentino “type” and Pit Bull “type”) subject to legislation (Table S1). We focused on responses from dog owners living in the UK and the Republic of Ireland. A very limited number of dog breeds/types is banned in the UK, and therefore we could obtain data on breeds legally owned in the UK but banned in other countries. We did not attempt to collect data from dog breeds/types banned in the UK, but our online questionnaire was open to the public without restriction, and therefore we obtained some data on banned dog breeds from the British Isles as well. The UK legislation bans Pit Bull “type” dogs, and we obtained too few entries to create a separate group for this breed type. However, we pooled data from three individuals described as Pit Bull “type” (of unspecified breed) and one Dogo Argentino individual together with other legislated breeds in a comparison of legislated versus non‐legislated types. The Staffordshire Bull Terrier is not banned in the UK, and was considered separately from the Pit Bull “type”.

In order to define non‐legislated breeds, the following criteria were used: (a) the breed was not classified as a legislated breed, (b) the breed did not belong to the same genetic cluster (based on Parker et al., 2017) as any of the legislated breeds (since it was assumed closely‐related breeds may show behavioural similarities resulting from common ancestry), and (c) data on the breed were available in our questionnaire database. With the exception of the breeds banned in the UK, all breeds had to be registered in the Kennel Club (UK). All dogs listed as crosses were excluded. Pedigree status could not be checked and so breed information is based solely on owner report. Breeds selected according to these criteria are listed in Table S1; the countries where different breeds are subject to legislation are listed in Table S2, and the details of the legislation are listed in Table S3.

In addition to comparing the legislated versus non‐legislated breed groups, we investigated differences between individual breeds. From the set of breeds used in that comparison, we selected breeds that had a sample size ≥20 individuals, with the exception of two legislated breeds – English Bull Terrier and Rhodesian Ridgeback. These two breeds were included in the breed‐level analysis despite smaller sample sizes due to their relevance to the primary research question. This resulted in 8 legislated breeds and 17 non‐legislated breeds used in the breed‐level analysis (Table 1).

**TABLE 1:** List of dog breeds meeting criteria for inclusion as a legislated or non‐legislated breed, which were used in breed‐level modelling, and the number of entries included for DIAS and PANAS analysis.

Breed	Legislation group	N (DIAS)	N (PANAS)
Akita	Legislated	65	73
Dobermann	Legislated	23	27
English Bull Terrier	Legislated	10	9
German Shepherd	Legislated	168	156
Great Dane	Legislated	28	32
Rhodesian Ridgeback	Legislated	13	13
Rottweiler	Legislated	54	57
Staffordshire Bull Terrier	Legislated	115	117
Beagle	Non‐legislated	30	35
Bearded Collie	Non‐legislated	21	22
Belgian Malinois	Non‐legislated	20	21
Border Collie	Non‐legislated	181	182
Border Terrier	Non‐legislated	24	23
Cavalier King Charles Spaniel	Non‐legislated	31	28
Cocker Spaniel	Non‐legislated	119	126
Dalmatian	Non‐legislated	30	32
Golden Retriever	Non‐legislated	84	87
Greyhound	Non‐legislated	95	91
Hungarian Vizsla	Non‐legislated	34	39
Labrador Retriever	Non‐legislated	188	175
Miniature Dachshund	Non‐legislated	23	25
Miniature Schnauzer	Non‐legislated	22	24
Springer Spaniel	Non‐legislated	80	76
West Highland White Terrier	Non‐legislated	31	30
Whippet	Non‐legislated	25	24

### 2.2 DIAS and PANAS questionnaires

The DIAS consists of 18 items rated on a 5‐point Likert scale: “strongly agree”, “agree”, “partly agree, partly disagree”, “disagree”, “strongly disagree”, with “not applicable” as the sixth option; 9 items are reverse scored. Values are summed and the total divided by five times the number of scored items to give an Overall Questionnaire Score (OQS) value between 0.2 and 1. The DIAS is composed of three factors: “Behavioural Regulation”, “Aggression Threshold and Response to Novelty” and “Responsiveness” (Wright et al., 2011). The scores for these factors were calculated from items that load on these factors according to Fadel et al. (2016). The PANAS is comprised of 21 items, and is scored in a similar way to the DIAS (Sheppard & Mills, 2002). This questionnaire is composed of two main factors: “Positive Activation” and “Negative Activation”.

Duplicates, blank entries, entries with four or more missing responses and those for dogs <1‐year‐old or owned by the current owner for less than a year were removed from the dataset. We also excluded responses from dog owners not based in the UK or Ireland, to reduce bias resulting from cultural or linguistic differences. We compared the DIAS and PANAS scores between legislated and non‐legislated groups (as defined above) and breed level differences for breeds with at least 20 individuals, with the exceptions noted in the previous section.

### 2.3 Statistical analysis

All statistical analyses were carried out in R V.4.1.0 (R Core Team, 2021) with the R code available in the Open Science Framework (https://osf.io/56neq/). Descriptive statistics are reported as means ± standard deviation unless otherwise stated, with final estimates rounded to 2 decimal places.

1845 DIAS entries were used for analysis: 506 individuals in the legislated group and 1339 individuals in the non‐legislated group. 1875 PANAS entries were used: 510 individuals in the legislated group and 1365 individuals in the non‐legislated group. Demographic information can be found in Table 2. The sample sizes for the breed‐level analysis were 476 individuals in the legislated group and 1038 individuals in the non‐legislated group for DIAS entries and 484 individuals in the legislated group and 1040 individuals in the non‐legislated group for PANAS entries.

**TABLE 2:** Demographic information for legislation status groups for the DIAS and PANAS data sets

Group	Gender	Neuter status	N (DIAS)	N (PANAS)
Legislated	Male	Entire	98	88
		Neutered	181	189
	Female	Entire	76	90
		Neutered	151	143
Total legislated			506	510
Non‐legislated	Male	Entire	237	232
		Neutered	504	516
	Female	Entire	186	203
		Neutered	412	414
Total non‐legislated			1339	1365
Total			1845	1875

For the DIAS data separate linear models were estimated due to assumption violations for MANOVA models relating to multivariate normality, outliers (156), as well as linearity and multi‐collinearity. To investigate differences in impulsivity between legislation status groups, four linear models for each DIAS variable were fitted as a function of legislation status, neuter status, sex, and all interaction effects. Analysis of Variance (ANOVA) tables were inspected and all *p* values adjusted for multiple comparisons with a Bonferroni correction. A threshold of *p* < 0.05 (after correction) was used for statistical significance. Partial eta squared (ηp^2^) effect sizes were calculated for factors in each linear model using the *lsr* package (Navarro, 2015) to quantify the unique variance each variable contributed to each psychometric response variable. Mean differences were also calculated, and, since some factors had slightly skewed distributions, confidence intervals were also estimated using a non‐parametric bootstrapping method with 2000 samples, based on the approach detailed by Thompson (2007). Common Language Effect Sizes (CLES) and Cohen's d were also calculated and are reported in the Supplementary Information.

To investigate whether there were differences in impulsivity at the breed level, other four linear models were fitted. However, given that we had no a priori reason to strongly hypothesise complicated interaction effects, and that the small sample sizes of some breeds meant only a single data point existed for some combinations of predictor variables (resulting in leverage warnings), we decided to estimate a simpler model with only the main effects of breed (25 levels), neuter status, and sex, without any interaction terms. *p* values were adjusted for the multiple comparisons as described above. Where breed was statistically significant (P_adj_ < 0.05) in the ANOVA tables, pairwise T‐tests using the pooled standard deviation across all breeds were conducted with the alpha level set at 1%, such that approximately 3 out of the 300 comparisons would be false positives. For all pairwise breed comparisons, Cohen's d was also estimated using the pooled standard deviation alongside 99% confidence intervals, such that approximately 3 out of 300 intervals would miss the true value. The Cohen D effect size information is reported in the Supplementary Figures. We were particularly interested in the DIAS variable “Aggression Threshold and Response to Novelty”, given its item content. Therefore, post hoc, we summed the number of statistically significant pairwise breed comparisons in this variable, in which: (1) a legislated dog breed scored higher than a non‐legislated dog breed, and (2) a non‐legislated breed scored higher than a legislated breed.

For the PANAS data, a Chi‐squared Q–Q plot showed only slight deviations from multivariate normality. However, there were 50 outliers present, and both the linearity and multi‐collinearity assumptions were violated. Therefore, the same approach as described above for DIAS was taken with the PANAS data set.

The data from all breeds pooled (i.e. the entire data set) were used for norm referencing. The mean score ±1 standard deviation (SD) is used to inform clinical judgement about the contribution of a trait to problem behaviour (Mills, in press). We therefore compared this range of each behavioural trait to the “typical range of scores” for a given breed, the latter defined as the range of scores between 25th and 75th percentile of the scores within the breed (i.e., the interquartile range).

## 3 RESULTS

**FIGURE 1:**
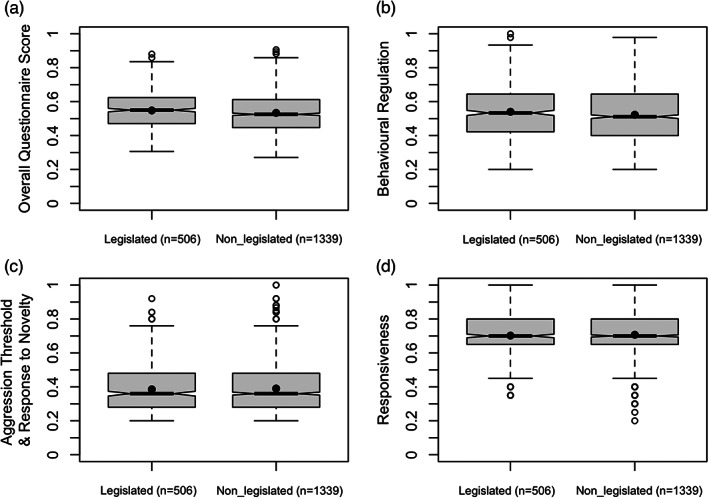
Boxplots of (a) *Overall Questionnaire Score*, (b) *Behavioural Regulation*, (c) *Aggression Threshold and Response to Novelty*, and (d) *Responsiveness*, between legislation status groups.

No significant differences were found between the legislated and non‐legislated group for DIAS OQS (mean difference = 0.01 [0.00, 0.03]), *Behavioural Regulation* (mean difference = 0.02 [0.00, 0.03]), *Aggression Threshold and Response to Novelty* (mean difference = 0.00 [−0.02, 0.01]), nor *Responsiveness* (mean difference = 0.00 [−0.02, 0.01]) (Figure 1, Table 3). The PCA plots showed no clustering of dogs from legislated vs non‐legislated groups for any of these three DIAS factors (Figures S1–S3). Furthermore, all estimated measures of difference (effect size) were small (see Table 4 for partial eta squared, and Table S5 for additional effect sizes). Neuter status was significant for *Aggression Threshold and Response to Novelty*, with neutered dogs scoring higher than non‐neutered dogs (0.40 ± 0.15 vs 0.37 ± 0.14). Neuter status was also significant for *Responsiveness*, with neutered dogs scoring lower than non‐neutered dogs (0.70 ± 0.13 vs. 0.72 ± 0.12). There were no significant interaction effects on any DIAS variable (Table S4).

**TABLE 3:** Descriptive statistics of DIAS and PANAS scores for each legislation status group and the total sample

	Mean ± SD	Median ± IQR
DIAS overall questionnaire score		
Legislated	0.548 ± 0.106	0.550 ± 0.153
Non‐legislated	0.534 ± 0.109	0.534 ± 0.165
Total	0.537 ± 0.109	0.529 ± 0.153
DIAS behavioural regulation		
Legislated	0.540 ± 0.160	0.533 ± 0.222
Not‐legislated	0.523 ± 0.165	0.511 ± 0.244
Total	0.527 ± 0.163	0.533 ± 0.244
DIAS aggression threshold and response to novelty		
Legislated	0.385 ± 0.152	0.360 ± 0.200
Non‐legislated	0.390 ± 0.149	0.360 ± 0.200
Total	0.389 ± 0.150	0.360 ± 0.200
DIAS responsiveness		
Legislated	0.702 ± 0.126	0.700 ± 0.150
Non‐legislated	0.707 ± 0.133	0.700 ± 0.150
Total	0.706 ± 0.131	0.700 ± 0.150
PANAS positive activation		
Legislated	0.677 ± 0.124	0.680 ± 0.195
Non‐legislated	0.667 ± 0.131	0.680 ± 0.180
Total	0.670 ± 0.129	0.680 ± 0.180
PANAS negative activation		
Legislated	0.476 ± 0.155	0.450 ± 0.220
Non‐legislated	0.481 ± 0.157	0.450 ± 0.220
Total	0.480 ± 0.157	0.450 ± 0.220

**TABLE 4:** Results from ANOVA for DIAS and PANAS variable linear models, including partial eta squared effect size (ηp^2^). Note that this table excludes the non‐significant interaction effects. The full table can be found in Tables S2 and S5.

	*F* statistic	P_adj_	ηp^2^
DIAS OQS			
Legislation status	*F*_1, 1837_ = 6.73	0.07	0.004
Neuter status	F_1, 1837_ = 2.17	0.99	0.001
Sex	*F*_1, 1837_ = 6.26	0.09	0.003
DIAS behavioural regulation			
Legislation status	*F*_1, 1837_ = 4.6	0.22	0.002
Neuter status	*F*_1, 1837_ = 1.78	1	0.001
Sex	*F*_1, 1837_ = 3.72	0.38	0.002
DIAS aggression threshold & response to novelty			
Legislation status	*F*_1, 1837_ = 0.21	1	0.000
Neuter status	*F*_1, 1837_ = 17.94	0.0002	0.010
Sex	*F*_1, 1837_ = 1.17	1	0.001
DIAS responsiveness			
Legislation status	*F*_1, 1837_ = 0.7	1	0.000
Neuter status	*F*_1, 1837_ = 15.51	0.0006	0.008
Sex	*F*_1, 1837_ = 0.57	1	0.000
PANAS positive activation			
Legislation status	*F*_1, 1867_ = 1.99	1	0.001
Neuter status	*F*_1, 1867_ = 35.47	<0.0001	0.019
Sex	*F*_1, 1867_ = 0.85	0.71	0.0005
PANAS negative activation			
Legislation status	*F*_1, 1867_ = 0.12	1	0.0001
Neuter status	*F*_1, 1867_ = 48.92	<0.0001	0.026
Sex	*F*_1,1867_ = 1.36	0.49	0.0007

Breed level analysis identified a statistically significant effect of breed for OQS (*F*_24, 1487_ = 3.22, P_adj_ <0.0001; ηp^2^ = 0.05), *Behavioural Regulation* (*F*_24, 1487_ = 3, P_adj_ < 0.0001; ηp^2^ = 0.05), *Aggression Threshold and Response to Novelty* (*F*_24, 1487_ = 5.65, P_adj_ <0.0001; ηp^2^ = 0.08), and *Responsiveness* (*F*_24, 1487_ = 8.12, P_adj_ <0.0001; ηp^2^ = 0.12). The PCA plots showed no clustering of dogs from the same breed for any of the three DIAS factors (Figures S4–S6). Neuter status was significant for *Aggression Threshold and Response to Novelty* (*F*_1, 1487_ = 10.47, P_adj_ = 0.0005; ηp^2^ = 0.01) and *Responsiveness* (*F*_1, 1487_ = 10.47, P_adj_ = 0.0037; ηp^2^ = 0.01), while sex had no statistically significant effects in any model (see Table S6 for all breed level ANOVA results).

**FIGURE 2:**
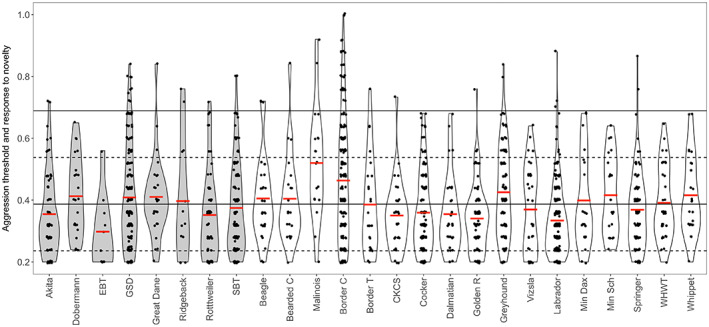
Violin plot of *Aggression Threshold and Response to Novelty* scores in the 25 breeds used in the breed level analysis. Each black dot within the box is an individual's score. The solid horizontal red line through each box represents each breed's mean. The solid horizontal line through the entire plot represents the total sample mean. The upper and lower dotted line represents ±1 SD from the mean, and the upper and lower solid line represents +2 SD from the mean (no lower solid line due to the lowest possible score being 0.2). [Correction added on 21 October 2022, after first online publication: Due to a production error, Figure 2 has been corrected in this version].

All but two dog breeds had their typical range of scores for the OQS within 1 SD of the reference mean (i.e. mean score for all breeds). The two exceptions were English Bull Terrier (legislated) and Beagle (non‐legislated); both having parts of their typical range of scores higher than the reference threshold (Figure S7). For *Aggression Threshold and Response to Novelty*, three breeds had their typical range of scores higher than reference threshold: Rhodesian Ridgeback (legislated), Belgian Malinois and Border Collie (both non‐legislated) (Figure 2). For *Behavioural Regulation*, one legislated breed (English Bull Terrier) had its typical range of scores higher than the reference threshold, and six non‐legislated breeds had their typical range of scores outside this threshold (Figure S8). For *Responsiveness*, one legislated breed (Rhodesian Ridgeback) had its typical range of scores higher than the reference threshold, another legislated breed (English Bull Terrier) had its typical range of scores lower than the reference threshold, and six non‐legislated breeds had their typical range of scores outside this threshold (Figure S9). For all the DIAS factors, the typical range of scores for all the breeds was within 2 SD of the global mean (i.e. within the statistical norm).

Pairwise comparison *T*‐tests (Figures S10–S13) revealed 30 differences between breeds in OQS, 38 in *Behavioural Regulation*, 40 in *Aggression Threshold and Response to Novelty*, and 68 in *Responsiveness*. For *Aggression Threshold and Responsive to Novelty*, of the 136 comparisons between a legislated and non‐legislated breed, 24 comparisons were statistically significant at the 1% alpha level. Of these, 8 involved a non‐legislated breed scoring higher than a legislated breed, and 15 involved a legislated breed scoring higher than a non‐legislated breed. Of the latter, 11 were due to the Belgian Malinois scoring significantly higher compared to a non‐legislated breed. Further, the non‐legislated Border Collie scored significantly higher than a legislated breed in 5 comparisons, and significantly higher than other non‐legislated breeds in 7 comparisons (see Figures S10–S13 for Cohen's d effect sizes). Interestingly, the primary involvement of the Belgian Malinois and Border Collie in significant *Aggression Threshold and Response to Novelty* comparisons was not mirrored for *Behavioural Regulation*, which represents a narrower assessment of impulsivity with features of high physiological arousal (Wright et al., 2011). For *Behavioural Regulation*, the Beagle was significantly higher in 9 comparisons, the Belgian Malinois only significantly higher in 1 comparison (vs Miniature Schnauzer), and the Border Collie only significantly higher in 3 comparisons (vs Golden Retriever, Labrador, and Miniature Schnauzer). With regards to *Responsiveness*, statistically significant comparisons involving the Akita (legislated), Great Dane (legislated), Whippet (non‐legislated), and Greyhound (non‐legislated) were consistently present (Suppl. Figure 13), with these breeds scoring particularly low (0.65 ± 0.12, 0.63 ± 0.12, 0.64 ± 0.15, and 0.60 ± 0.14 respectively) for this DIAS variable, for which the mean breed level for the data set was 0.71 ± 0.13 (Figure S9). The majority of Cohen's d effect sizes for all DIAS variables had large confidence intervals, and as such, there is considerable uncertainty surrounding the point estimate of the size of these breed differences.

In the PANAS analysis, legislation status had no effect on either *Positive Activation* (mean difference = 0.01 [0.00, 0.02]) or *Negative Activation* (mean difference = 0.00 [−0.02, 0.01]) (Tables 3 and 4; see Table S5 for effect sizes and Figure S14 for boxplot). The PCA plot showed no clustering of dogs from legislated vs non‐legislated groups for these two PANAS factors (Figure S15). Neuter status had very small but statistically significant effects on both *Positive Activation* (neutered = 0.66 ± 0.13, entire = 0.70 ± 0.12), and *Negative Activation* (neutered = 0.50 ± 0.16, entire = 0.44 ± 0.15), whereas sex had no effect on either of these (Table 4). The factorial ANOVA analysis did not detect any interaction effects for either *Positive Activation* or *Negative Activation* (Table S7).

Breed level models of the PANAS data (Table S8) detected statistically significant main effects of breed for both *Positive Activation* (*F*_24, 1509_ = 11.36, P_adj_ <0.0001; ηp^2^ = 0.15) and *Negative Activation* (*F*_24, 1509_ = 2.86, P_adj_ <0.0001; ηp^2^ = 0.04). The PCA plots showed no clustering of dogs from the same breed for the two PANAS factors (Figure S16). Neuter status affected both *Positive Activation* (*F*_1, 1509_ = 15.24, P_adj_ = 0.0002; ηp^2^ = 0.001) and *Negative Activation* (*F*_24, 1509_ = 32.14, P_adj_ <0.0001; ηp^2^ = 0.02), but sex had no effect (*Positive Activation*: *F*_1, 1509_ = 1.25, P_adj_ = 0.53; ηp^2^ = 0.0008; *Negative Activation*: *F*_1, 1509_ = 0.92, P_adj_ = 0.67; ηp^2^ = 0.0006).

**FIGURE 3:**
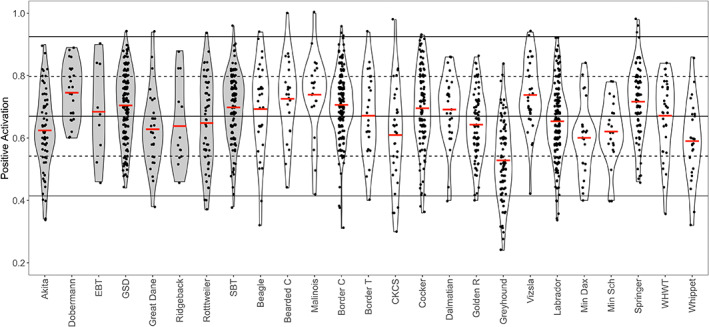
Violin plot of PANAS *Positive Activation* in the 25 breeds used in the breed level analysis. Each black dot within the box is an individual's score. The solid horizontal red line through each box represents each breed's mean. The solid horizontal line through the entire plot represents the total sample mean. The upper and lower dotted line represents ±1 SD from the mean, and the upper and lower solid line represents ±2 SD from the mean.

For *Positive Activation*, two legislated breeds (Dobermann and English Bull Terrier) had their typical range of scores higher than the reference threshold (mean ± 1 SD), and seven non‐legislated breeds had their typical range of scores outside the reference threshold (Figure 3). For *Negative Activation*, one legislated breed (Rhodesian Ridgeback) had its typical range of scores lower than the reference threshold, and three non‐legislated breeds had their typical range of scores outside the reference threshold (Figure S5). For both *Positive Activation* and *Negative Activation*, the typical range of scores for all the breeds was within 2 SD of the reference mean (statistically normal range).

Pairwise comparisons for *Positive Activation* (Figure S17) revealed 112 significant comparisons at the 1% alpha level. Of note, Greyhound (non‐legislated) had significantly lower scores than 23 other breeds, and their mean score was 0.53 ± 0.12 compared to the overall mean of 0.67 ± 0.13. The Akita (legislated) who also had a low *Positive Activation* mean (0.62 ± 0.12) was significantly lower than 10 other breeds. Breeds that were involved in several comparisons involving scoring significantly higher on *Positive Activation* included the Dobermann (legislated; higher than 7 non‐legislated breeds and 4 legislated breeds), Belgian Malinois (legislated; higher than 7 non‐legislated breeds and 3 legislated breeds), Springer Spaniel (non‐legislated: higher than 7 non‐legislated breeds and 3 legislated breeds), Hungarian Vizsla (non‐legislated: higher than 7 non‐legislated breeds and 4 legislated breeds), Bearded Collie (non‐legislated: higher than 7 non‐legislated breeds and 3 legislated breeds), and Border Collie (non‐legislated: higher than 7 non‐legislated breeds, and 3 legislated breeds). There were 24 statistically significant differences in *Negative Activation* at the 1% alpha level. Of note, 11 of these involved the Border Collie scoring higher, which included four comparisons to legislated breeds (Rhodesian Ridgeback, Rottweiler, German Shepherd, and Akita). Further, 9 comparisons involved the Staffordshire Bull Terrier (legislated) scoring higher, including 4 comparisons against legislated breeds and 5 against not‐legislated breeds. Similar to DIAS, Cohen's d effect sizes for PANAS pairwise comparisons generally had wide CIs, meaning that for the majority of comparisons there is considerable uncertainty regarding the size of breed‐related differences (Figures S17 and S18).

## 4 DISCUSSION

### 4.1 DIAS and PANAS factors as predictors of the tendency towards aggressive behaviour in dogs – Advantages and limitations

Dog Impulsivity Assessment Scale is an extensively validated psychometric instrument developed to measure trait level impulsivity in dogs (Brady et al., 2018; Fadel et al., 2016; Riemer et al., 2013; Wright et al., 2011, 2012). Trait level impulsivity relates to the aspect of the executive control of behaviour, which measures the degree to which an individual responds to stimuli in the environment with or without wider appraisal. DIAS scores have been shown to be stable within subjects over a time frame of several years (Riemer et al., 2013). Moreover, the clustering of items into three factors has been replicated in two populations independent of the original study population (Fadel et al., 2016 and the present study). The overall questionnaire score as well as the three composite factors have been shown to correlate with the outcomes of two different experimental behavioural tests that measure impulsivity (a delayed reward test – Wright et al., 2012 and spatial discounting task – Brady et al., 2018). The questionnaire scores have also been shown to correlate with levels of serotonin and serotonin/dopamine ratio from urine samples (Wright et al., 2012). These systems are associated with problematic aggression in dogs at both the genetic level (Våge et al., 2010) and phenotypically (e.g. Amat et al., 2013; Peremans et al., 2003).

Positive and Negative Activation Scale (Sheppard & Mills, 2002) is a longer standing instrument originally developed on the basis of the now widely accepted concept of core affect to animal emotions (Mendl et al., 2010). In its development (Sheppard & Mills, 2002), key attributes of the scale relating to test–retest reliability, internal consistency and the unidimensionality of the two traits relating to positive and negative affect were established. Later validation (Sheppard & Mills, 2003) focused initially on convergence with clinically relevant populations (dogs with noise fears) and their response to treatment, consistent with predictions from the equivalent human scale (Watson et al., 1988). Subsequent work has further demonstrated convergence with a range of behavioural (Brady et al., 2018), psychological (McPeake & Mills, 2017) and physical states (e.g. Reaney et al., 2017) associated with relevant psychological states. The scale has shown convergent validity with relevant clinical outcomes (McPeake & Mills, 2017) and its structure has demonstrated good stability across other cultures (Savalli et al., 2019).

There are also other data showing that psychometric tools can correctly predict aggressive tendencies in dogs. For example, strong positive correlation was found between all the components of C‐BARQ questionnaire (Hsu & Serpell, 2003) related to aggression and fear (stranger‐, owner‐, dog‐ and familiar dog‐directed aggression, stranger‐directed, dog‐directed and non‐social fear) and veterinary behavioural diagnoses in 122 dogs (Zapata et al., 2022). Moreover, several genetic loci have been shown to have a significant association with these behavioural traits (Zapata et al., 2016) and could correctly predict individual behaviour in a cohort of nearly 400 pure‐ and mixed‐breed dogs, while a subset of those loci could predict veterinary behavioural diagnoses (Zapata et al., 2022). This demonstrates that owner‐assessed behavioural traits can have a strong genetic component and may serve as good predictors of individual behavioural problems. Nevertheless, we do not have direct evidence that any of the DIAS or PANAS factors is a strong predictor of the risk of publicly reported attacks.

Although behavioural questionnaires have the advantage of enabling the rapid collection of data on a large number of dogs from a broad spectrum of owners, they are also associated with some limitations. First, the assessment of dog behaviour by its owner may be biased, especially in the assessment of problem behaviours such as aggression. This bias is intrinsic to all behavioural questionnaires, including those developed for humans. However, the development of DIAS and PANAS sought to minimise this bias by asking questions about reactions to standard every‐day situations that do not include any judgement on expected vs “problem” behaviours. Correlation of the questionnaire‐based assessment with the outcomes of experimental behavioural tests (Brady et al., 2018; Wright et al., 2012), neurotransmitter levels in urine samples (Wright et al., 2012) and clinical outcomes (McPeake & Mills, 2017) testifies to the reliability of DIAS and PANAS. In the current study, dog owners were informed that we were carrying out research into behavioural differences between breeds, but were not specifically informed about the primary research aim, which further minimised the risk of differential bias between owners of different breeds concerning the questions which assessed the tendency towards aggression.

The second potential type of bias relates to the data not originating from a random sample of all representatives of each breed studied, i.e. self‐selection bias amongst respondents. It is possible that dog owners' willingness to participate may be related to their dogs' behavioural tendencies. This bias, however, also applies (arguably to a larger extent) to other methods of behaviour assessment, such as experimental behavioural tests or analyses of clinical data. Filling in a questionnaire requires less time and dedication from a dog owner than any form of direct assessment of behaviour by a specialist, and therefore it is likely that a broader range of dog owners can be reached. In this study, we used social media to target owners and breeders of the dog breeds of interest within the UK and the Republic of Ireland. Large sample sizes obtained (albeit not for all breeds) as well as the fact that we also received responses from non‐targeted audience (dog owners living in other countries as well as owners of breeds banned in the UK) testify to the effectiveness of this approach in reaching a broad range of participants. The absence of a minority of dogs who might represent an extreme within the breed, does not undermine our primary result that highlights the variability within the breed of the traits examined.

The third potential bias relates to the use of breeds from a “legislated group”, which are under legislative control in some countries (see Table S2), but not in the UK where the survey was done. Given that breeding of individuals from the same breed is considerably more frequent within each country than between countries, and different sets of behavioural traits may be selected in different countries, representatives of the same breed from different countries may potentially differ in behaviour. However, genomic analyses of differentiation within versus among breeds do not provide evidence for differentiation between representatives of the same breeds from different countries, at least within Europe. For example, individuals belonging to the same breed sampled in the UK (Pilot et al., 2015) and continental Europe (Vaysse et al., 2011) were shown to cluster together in a pulled dataset (Pilot et al., 2015). This shows that the gene flow between countries is sufficient to prevent genetic differentiation within breeds, and therefore differences in heritable behavioural traits are unlikely. Environmental factors such as dog training practices might also result in differentiation of behavioural traits between representatives of the same breed in different countries. This is, however, beyond the scope of this study.

### 4.2 Comparison of behavioural tendencies between legislated and non‐legislated breeds

Our results indicate that while there are differences between specific breeds of dogs in some of the traits that might affect the risk of aggressive behaviour, this is not consistently related to the legislative status of these breeds. With regards to breeds legislated against in some countries, the English Bull terrier and Rhodesian Ridgeback appear to have the distribution of scores for several traits (especially in relation to impulsivity, but also some aspect of positive and negative affect) that might make them higher risk breeds. It is important to note, however, that these breeds had the smallest sample sizes. Moreover, several breeds which are not legislated against in the UK or elsewhere have similar trait scores, in particular the Border Collie. This finding is in accordance with studies showing that Border Collies are among the most frequently reported breeds involved in bite incidents in both the UK (Oxley et al., 2018) and Republic of Ireland (Creedon & Ó'Súilleabháin, 2017), although it should be noted that they are a popular breed and therefore overrepresented in the general dog population (Oxley et al., 2018). When accounting for population prevalence, German Shepherds, Rottweilers and “terriers” have all been implicated in separate studies as the types most commonly involved in dog bite incidences (Morgan et al., 2017). The non‐legislated Belgian Malinois had the highest average score of all breeds examined in the primary trait of interest (*Aggression Threshold and Response to Novelty*), and was detected as scoring higher in 11 of the 24 statistically significant pairwise comparisons for this score. This may reflect the widespread use of this breed for protection work; aggression in the face of novelty may be a desirable attribute in such circumstances, although this breed did not present with consistently higher *Negative Activation* scores in the pairwise comparisons in the current analysis.

The majority of pairwise breed comparisons did not yield significant differences. There were few significant pairwise differences between legislated and non‐legislated breeds, and the majority of significant inter‐breed differences found in the primary trait of interest (*Aggression Threshold and Response to Novelty*) were differences between pairs of non‐legislated breeds. The non‐legislated Border Collie scored significantly higher in *Aggression Threshold and Response to Novelty* compared to 8 other breeds. Despite having the highest average score, the Belgian Malinois only scored statistically higher than the Labrador Retriever. It should be noted that breed sample sizes varied markedly, with some samples being relatively small, including that of the Belgian Malinois (DIAS = 20, PANAS = 21), and so the potential effect of sampling bias must be considered. Small sample sizes can reduce the power to detect significant differences; to some degree, this issue can be mitigated by examining the clinically relevant difference (i.e. differences of more than 1 SD from the population mean), but this will not control for biased sampling.

Effect size measures for the main effect of breed ranged from 0.04 to 0.15, indicating that a large proportion of variation in DIAS and PANAS scores is not attributable to breed, and thus breed is a poor predictor of individual behavioural predispositions. In particular, large variation in the trait *Aggression Threshold and Response to Novelty* suggests that individual dogs cannot be classified as “dangerous” or “safe” solely based on their breed. Large variation within breeds has also been demonstrated for a broad range of other canine behavioural traits, with breed explaining only 9% of variation in behaviour (Morrill et al., 2022). Large variation of behavioural traits within breeds may be the product of recent relaxation of selection on behavioural traits in breeds, with breed standards and breed success in competitions being focused mainly on morphological traits. Alternatively, it may suggest that the behavioural traits assessed within this study were never subject to strong selection within breeds, although we consider this unlikely given the working history of many breeds. Indeed, a comparison of impulsivity levels, based on DIAS questionnaire scores, between work and show lines of Border Collies and Labrador Retrievers, showed that working Collies were significantly more impulsive, on average, compared to working Labradors, but show lines from the two breeds were not significantly different (Fadel et al., 2016). Therefore, it is possible that the partial or complete loss of original work function (e.g. as guarding, hunting and herding dogs) as a basis for selection in many breeds led to the reduction of behavioural differences between them.

We found that for all DIAS and PANAS factors the typical range of scores, defined as the range between 25th and 75th percentile of the scores within a breed, was within ±2 SD range from the reference mean for all the breeds considered, independent of their legislation status. This indicates that no breed investigated here scores extremely high or low on any of the psychometric variables assessed here. Although our results imply that specific breeds may differ *on average* in their tendency towards aggressive behaviour, it is worth noting that not only was the average score for *Aggression Threshold and Response to Novelty* not significantly different between the groups of legislated vs non‐legislated breeds, but within both groups about half of the breeds had mean scores below the total population mean (4/8 breeds subject to legislation and 7/17 non‐legislated breeds). We could find no evidence that the legislated breeds as a group were differentiated from the non‐legislated breeds on the basis of the behavioural traits related to the risk of aggressive behaviour, which were assessed in this study.

The comparison of the PANAS results between breeds also suggests that the dogs that become easily excited may be perceived as more dangerous. Two legislated breeds, Dobermann and English Bull Terrier, which are commonly considered to be “aggressive” by the public (Clarke et al., 2016; Mills et al., 2013), had their typical range of scores for the PANAS factor *Positive Activation* higher than the reference threshold, although this also applied to several non‐legislated breeds (Figure 3). Dobermanns have previously been reported to have high scores for the C‐BARQ factor “energy” (Serpell & Duffy, 2014). Dogs with particularly high scores for *Positive Activation* can be considered to be more energetic and excitable (Sheppard & Mills, 2002), which could potentially lead to frustration when they are denied access to something that they desire. This may be demonstrated by the dog pulling on the lead whilst barking, which may appear ‘aggressive’ or cause for concern to an individual unfamiliar with dog behaviour (Shepherd, 2017). Certain breeds, such as the Dobermann and English Bull Terrier, are more likely to be perceived as dangerous when lunging and barking than, for example, a Golden Retriever, regardless of the underlying emotional state that is driving the behaviour (Clarke et al., 2016). This could potentially lead to the perception that these breeds are more likely to exhibit aggressive behaviour.

Behavioural differences between dog breeds and individual dogs are influenced by complex interactions between genetic and environmental factors that are poorly understood (Fadel & Pilot, 2017; Spady & Ostrander, 2008). Dog breeds have been developed under selective pressure based on the role they are required to fulfil (Parker et al., 2017). Artificial selection during breed formation resulted in the fixation of certain morphological traits within breeds, many of which have a known genetic basis (Ostrander et al., 2017). Our analysis of behavioural tendencies measured using the DIAS and PANAS questionnaires showed that the behavioural traits assessed within this study are unlikely to be fixed within breeds, displaying instead a large variation within each breed assessed. Although we found significant differences in behavioural tendencies between some pairs of breeds, implying that breeds differ in their typical behaviour, the high level of variability means that the behavioural tendencies of individual representatives of a breed may differ considerably from breed‐typical behaviour. Therefore, breed is a poor predictor of the behavioural tendency of the individual.

This variability may reflect varying selective pressures acting on dog populations, which may result in highest fitness for intermediate behavioural phenotypes or facilitate the maintenance of multiple phenotypes. Dogs have steeper dominance hierarchy in conspecific groups than wolves (Range et al., 2015), and social tolerance towards other dogs and humans is associated with low status in dominance hierarchy (Vékony et al., 2022). At the same time, in the case of pure‐bred dogs whose reproduction is controlled by humans, individuals showing higher social tolerance and a lower tendency towards aggression are more likely to be selected for breeding, and therefore these traits may be maintained in dog populations.

Alternatively, intra‐breed variation in behavioural traits may result from limited ongoing artificial selection pressure on maintaining certain behavioural traits in breeds, given that breed standards and show performance are mostly focused on morphological traits. Therefore, popular breeds are likely to be bred with limited consideration of health issues or behavioural traits. Working dogs may be an exception, and indeed working dog lines have been shown to display larger inter‐breed behavioural differences than lines of pet or show dogs (Fadel et al., 2016). This might explain why the Border Collie stands out in breed comparisons within our sample, since it was only registered with the Kennel Club in the mid 1970s, prior to which the standard was focused on working ability.

### 4.3 Behavioural tendencies as risk predictors of dog attacks

Although owner‐based psychometric scaling results are used clinically to assess risk, there is little published research relating these results to specific risk outcomes such as the probability of a bite incident or its severity. With regard to the latter, it should be noted that large breeds such as German Shepherds are more likely to cause more extensive injuries compared to smaller breeds (Wake et al., 2009) even if the pattern and intensity of aggression are exactly the same. Thus, based on the injuries resulting from the attack, a large dog can be perceived as more dangerous even without having higher aggressive tendencies. For example, Pit Bulls were shown to have a prevalence of human‐directed bites only slightly above the average, but the severity of their attacks may be affected by their size and strength (Duffy et al., 2008). Moreover, perceptions of dangerousness may be stronger in the case of legislated dog breeds, and may be based on the reputation of both these breeds and their typical owners (Clarke et al., 2016; Harding, 2014).

Several studies point to an association between ownership of “high‐risk” dogs and the presence of antisocial behaviour in the owners as indicated by criminal convictions, as well as specific personality traits of the owners such as sensation seeking and primary psychopathy (Barnes et al., 2006; Harding, 2014; Ragatz et al., 2009). The studies also point to a high correlation between violence against humans and animals (Barnes et al., 2006), potentially implying that dog owners involved in aggressive crimes are more likely to mistreat dogs they own and train them to act aggressively. A mistreated dog is more likely to act aggressively than a dog who is treated adequately (Barnes et al., 2006). For example, among Pit Bulls seized in investigations of organized dog fighting, a significant correlation was found between the amount of scarring present and aggression towards other dogs (Miller et al., 2016). This is consistent with a history of involvement in dog fights and presumably training for aggressive behaviour against other dogs leading to an increased tendency towards aggression (Miller et al., 2016). Another study showed that dogs adopted from shelters were significantly more likely to show defensive aggression towards their owners compared with dogs obtained from other sources, suggesting that their behaviour could have been affected by negative prior experiences (Notari et al., 2020).

Therefore, many individual dogs within banned breeds may act aggressively not because of their intrinsic higher than average aggressive tendency, but because they more frequently have owners who mistreat them and/or specifically train them to act aggressively. This may lead to a correlation between “dangerous breeds” and aggressive behaviour that may be independent of the natural aggressive tendencies in these breeds. Accordingly, Notari et al. (2020) concluded that overrepresentation of some breed groups among dogs referred to veterinary behaviourists by public authorities in Italy due to an incidence of aggression may result from stereotyped perceptions of some breeds, which increased the probability of reporting to authorities. All overrepresented breeds/breed types in that study – German Shepherd, Rottweiler, Dogo Argentino, Bull Terrier, American Staffordshire Terrier, Pit Bull – are subject to legislation in some countries (Table S1). Both the relationship between psychometric score and the risk of aggression as well as factors predicting the severity of attack are important areas for future research, as they relate to different types of risk, which should not be conflated.

## 5 CONCLUSIONS AND PRACTICAL IMPLICATIONS

Taken together, our findings indicate that breed alone is not a reliable predictor of individual behavioural tendencies for the traits examined here. The lack of consistent clinically or statistically significant differences, alongside small effect sizes between legislated and non‐legislated breeds and types, combined with large intra‐breed variation for these traits challenge the scientific soundness of breed specific legislation. Accordingly, we suggest legislation that focuses on individual behavioural tendencies (e.g. Control of Dogs [Scotland] Act 2010) is likely to be more effective at maintaining public safety.

## CONFLICT OF INTEREST

The authors declare no conflicts of interest.

## Supporting information


Supporting information S1

## Data Availability

Data for this study is available at the Dryad Digital Repository (Hammond et al., 2022; https://doi.org/10.5061/dryad.ffbg79cz9). Benefits from this research result from the public sharing of our data and results.
