# Supplementary material for: Comparison of behavioural tendencies between “dangerous dogs” and other domestic dog breeds – Evolutionary context and practical implications
Source: Evol Appl. 2022 Oct 19;15(11):1806–19. doi: 10.1111/eva.13479 (PMC9679229; doi:10.1111/eva.13479)
Supplement: Supplementary file 1 — Supporting information S1 [file EVA-15-1806-s001.pdf]

Comparison of behavioural tendencies between “dangerous dogs” and other domestic dog breeds – evolutionary context and practical implications

Alexa Hammond, Thomas Rowland, Daniel S. Mills & Małgorzata Pilot

Supplementary Information

Validation of DIAS and PANAS

This study allowed for further validation for both DIAS and PANAS, with larger sample sizes than the original studies developing these questionnaires (Sheppard and Mills, 2002; Wright et al., 2011). The DIAS development paper considered loadings $\geq$ 0.4 as high, and the PANAS development paper considered loadings $\geq$ 0.35 as high. Here, we report all loadings $\geq$ 0.35. In general, The PCA results were similar to those of the original studies. The DIAS structure remained intact with three exceptions where cross-loadings were identified. Item 1 “My dog shows extreme physical signs when excited (e.g. drooling, panting, raising hackles, urination, licking lips, widening of eyes)” loaded on to Behavioural Regulation (0.459) as originally, but also Responsiveness (0.375, but this is under the DIAS threshold used in development). Item 10 “My dog is easy to train” loaded on to Responsiveness (0.510) like the original paper, but also cross-loaded on to Behavioural Regulation (-0.479). Item 15 “My dog is very interested in new things and new places” loaded onto the Aggression Threshold and Novelty component (-0.6) as it did originally, but here it also cross-loaded on to Responsiveness (0.491). Also, item 6 “My dog appears to be ‘sorry’ after it has done something wrong” loaded on to Responsiveness (0.349) if a 0.35 threshold were chosen and rounded to two decimal places, or it did not load on to any component if a 0.4 threshold were chosen. Some of these differences are in line with prior work (Fadel et al., 2016). We therefore used the scoring as in Fadel et al. (2016) whereby item 6 was removed, and item 10 was only included in the calculation of factor 3. Of note, PCA scores all correlated extremely highly with the calculations used (all > 0.87).

The PANAS structure also showed several differences. Item 9 “Your dog tries to escape from the garden” did not load onto any factor, and item 18 “Your dog persists in being naughty despite being told off for the behaviour” did not load on to either principal component. Both PCA scores from the two-component solution from the current dataset correlated extremely highly with the scoring system used in the original paper (both >0.96). For this paper, we therefore used the original scoring for positive activation and negative activation as reported in Sheppard and Mills (2002).

Supplementary Tables

Supplementary Table S1. List of dog breeds meeting criteria for inclusion as a legislated or non-legislated breed along with the genetic clades they belong to as reported by Parker et al. (2017).

Legal status	Breeds	Genetic clade
Legislated in some countries	Akita and Tibetan mastiff	Asian Spitz
	Bull terrier, Bullmastiff, Dogue de Bordeaux, Great Dane, Neapolitan mastiff, Rhodesian Ridgeback and Staffordshire Bull Terrier	European Mastiff
	Dobermann and Rottweiler	Drover
	German Shepherd	“New World”
	Dogo Argentino “type” and Pit bull “type”	Not listed in a genetic clade
Non-legislated	Australian cattle dog, Australian shepherd, Bearded Collie, Border Collie, Cardigan Welsh Corgi, Deerhound, Greyhound, Italian Greyhound, Old English Sheepdog, Pembroke Welsh Corgi, Shetland Sheepdog and Whippet	UK Rural
	Belgian Shepherd Malinois and Belgian Shepherd Tervuren	Continental Herder
	Cavalier King Charles Spaniel, English Cocker Spaniel, and English Springer Spaniel	Spaniel
	Flatcoated Retriever, Golden Retriever, Irish Water Spaniel, Labrador Retriever, Newfoundland, and Nova Scotia Duck Tolling Retriever	Retriever
	Brittany, Dalmatian, English Setter, Gordon Setter, German Shorthaired Pointer, German Wirehaired Pointer, Hungarian Vizsla, Irish Setter, Irish Red and White Setter, and Weimaraner	Pointer & Setter
	Bassett Hound, Beagle, Dachshund and Miniature Dachshund	Scent Hound
	Bedlington Terrier, Border Terrier, Cairn Terrier, Parson Russell Terrier, Norfolk Terrier, Norwich Terrier, Scottish Terrier, West Highland White Terrier and Yorkshire Terrier	Terrier
	Bichon Frise, Havanese, Maltese, Miniature Poodle, Standard Poodle and Toy Poodle	Poodle
	Miniature Schnauzer	Schnauzer
	Lhasa Apso, Shih Tzu and Tibetan Terrier	Asian Toy

Supplementary Table S2. List of dog breeds meeting criteria for inclusion as a legislated breed, for which we obtained the DIAS and PANAS questionnaire data in the current study, with the number and the list of countries where they are banned or subject to regulations.

Breed	N Countries	Countries where the breed is banned or regulated
Pit Bull*	26	Australia, Belarus, Bermuda Islands, Canada, Denmark, Ecuador, France, Germany, Ireland, Israel, Italy, Malta, New Zealand, Norway, Poland, Portugal, Romania, Russia, Singapore, Spain, Switzerland, Turkey, Ukraine, United Kingdom, USA, Venezuela
Staffordshire Bull Terrier	15	Bermuda Islands, Canada, France, Germany, Iceland, Ireland, Israel, Portugal, Singapore, Spain, Switzerland, Thailand, Ukraine, United Arab Emirates, USA
Rottweiler	15	Belarus, Bermuda Islands, France, Germany, Italy, Israel, Malaysia, Poland, Portugal, Singapore, Spain, Switzerland, Ukraine, United Arab Emirates, USA
Dogo Argentino*	15	Australia, Bermuda Islands, Israel, Italy, Malta, New Zealand Norway, Portugal, Romania, Singapore, Spain, Turkey, Ukraine, United Kingdom, USA
Dobermann	10	Belarus, Bermuda Islands, Ireland, Italy, Germany, Malaysia, Singapore, Ukraine, United Arab Emirates, USA
German Shepherd	7	Belarus, Bermuda Islands, Ireland, Malaysia, Singapore, Ukraine, USA
Rhodesian Ridgeback	4	Bermuda Islands, Ireland, Ukraine, USA
Great Dane	2	Belarus, Ukraine

*For these breeds/breed types we obtained very small sample sizes and therefore they were only analysed in the comparisons between legislated and non-legislated groups, but not at the level of individual breeds.

Supplementary Table S3. Type of legislation implemented in different countries for the legislated dog breeds that were assessed individually in the current study.

Breed	Country	Legislation
Dobermann	Belarus	A large number of breeds are considered dangerous and are banned
	Bermuda	Restricted
	Germany - Brandenburg state	State-wise banned breeds. Can be issued with an official authorisation to keep the dog
	Ireland	Restricted – must be handled by someone over 18 whilst being muzzled and on 2m lead in public, must have a collar with owner info, be licenced and microchipped
	Italy	Importation is restricted. Restrictions in the city of Venice. Italy repealed this breed specific legislation in 2009 and now the new legislation focused on incidents of aggressive behaviour.
	Malaysia	Restricted - Criteria to import. Must be a pet and not for sale. Secured at home. In public on lead and muzzled. Must be handled by a trained and skilled handler
	Singapore	Restricted - Can travel. Must undergo obedience training, on lead and muzzled in public
	Ukraine	Listed as a dangerous dog breed (Dobermann Pinscher) / Recognised as potentially dangerous (Dobermann)
	United Arab Emirates	Prohibited - cannot be imported into the country. All pet dogs vaccinated, registered, on lead in public with large dogs muzzled
	United States	Regulations depend on the state and city
German Shepherd	Belarus	A large number of breeds are considered dangerous and are banned
	Bermuda	Restricted
	Ireland	Restricted – must be handled by someone over 18 whilst being muzzled and on 2m lead in public, must have a collar with owner info, be licenced and microchipped
	Malaysia	Restricted - Criteria to import. Must be a pet and not for sale. Secured at home. In public on lead and muzzled. Must be handled by a trained and skilled handler
	Singapore	Restricted - Can travel. Must undergo obedience training, on lead and muzzled in public
	Ukraine	Listed as a dangerous dog breed
	United States	Regulations depend on the state and city
Great Dane	Belarus	A large number of breeds are considered dangerous and are

		banned
	Ukraine	Recognised as potentially dangerous
Rhodesian Ridgeback	Bermuda	Restricted
	Ireland	Restricted – must be handled by someone over 18 whilst being muzzled and on 2m lead in public, must have a collar with owner info, be licenced and microchipped
	Ukraine	Listed as a dangerous dog breed
	United States	Regulations depend on the state and city
Rottweiler	Belarus	A large number of breeds are considered dangerous and are banned
	Bermuda	Restricted
	France	Category 2 banned breed (pedigree or not) - Licence required and granted after a behavioural assessment has been completed by a veterinarian. Owners to complete an approved training course. Insurance. Dogs must be vaccinated and neutered/spayed. On lead and muzzled at all times in public
	Germany - Bavaria, Brandenburg, Hamburg and Hesse states	State-wise banned breeds. Can be issued with an official authorisation to keep the dog
	Italy	Importation is restricted. Restrictions in the city of Venice. Italy repealed this breed specific legislation in 2009 and now the new legislation focused on incidents of aggressive behaviour.
	Israel	Restricted - there are conditions to keeping this breed, which include being muzzled when a minor (< 16 years old) is in the vicinity, being handled by someone over 18 whilst being muzzled and on lead in public, neutering/spaying by 6 months of age and living in a secure location with clear signage to indicate that a dangerous dog is present.
	Malaysia	Restricted - Criteria to import. Must be a pet and not for sale. Secured at home. In public on lead and muzzled. Must be handled by a trained and skilled handler
	Poland	Restricted. Permit required
	Portugal	Restricted. A licence required (owners must be 18+, without criminal convictions, must have insurance and confirmed housing, security and behavioural history of the dog), which places several responsibilities on the licencee (e.g. the dog has to be always on lead and harness in public, lead must be under 1m in length, must attend socialisation classes with a certified trainer)
	Singapore	Restricted - Can travel. Must undergo obedience training, on lead and muzzled in public

	Spain	Restricted - Have to be registered, vaccinated, have a license and microchip, insurance, be on lead and muzzled in public, must be handled by a person 18+ years old, cannot escape property, lost/stolen to be reported immediately
	Switzerland	Restricted – must undergo a behavioural test, must be neutered, must have a lead and muzzle in public, and be handled by a person over 18 years old, must have an insurance and be a single pet in a household. Note that this varies depending on canton
	Ukraine	Listed as a dangerous dog breed
	United Arab Emirates	Prohibited - cannot be imported into the country. All pet dogs vaccinated, registered, on lead in public with large dogs muzzled
	United States	Regulations depend on the state and city
Staffordshire bull terrier	Bermuda	Restricted
	Canada	Depends on the province. Prohibited from entering or transiting in the province (Ontario, Manitoba). If they were being kept prior to 2005 they have to be spayed/neutered and on lead and muzzled in public
	France	Category 1 banned breed if no pedigree papers - Cannot import, buy or sell. Must neuter/spay, on lead and muzzled in public with no access to certain public places.<br>Category 2 banned breed if they are a pedigree.<br>For both categories a licence is required and granted after a behavioural assessment has been completed by a veterinarian. Owners to complete an approved training course. Must have insurance. Dogs must be vaccinated and neutered/spayed. On lead and muzzled at all times in public
	Germany	Prohibited - cannot be imported
	Iceland	Prohibited - cannot be imported
	Ireland	Restricted – must be handled by someone over 18 whilst being muzzled and on 2m lead in public, must have a collar with owner info, be licenced and microchipped
	Israel	Restricted - there are conditions to keeping this breed, which include being muzzled when a minor (< 16 years old) is in the vicinity, being handled by someone over 18 whilst being muzzled and on lead in public, neutering/spaying by 6 months of age and living in a secure location with clear signage to indicate that a dangerous dog is present.
	Portugal	Restricted. A licence required (owners must be 18+, without criminal convictions, must have insurance and confirmed housing, security and behavioural history of the dog), which places several responsibilities on the licencee (e.g. always on lead and harness in public, lead must be under 1m in length, must attend socialisation classes with a certified trainer)

	Singapore	Banned - prohibited from entering the country. If already in the country - neutered/spayed, on lead and muzzled in public, undergo obedience training
	Spain	Restricted - registered, vaccinated, licence and microchip, insurance, on lead and muzzled in public, cannot escape property, lost/stolen to be reported immediately, has to be walked by someone over 18 years of age
	Switzerland	Restrictions apply which vary depending on the canton. Restrictions may include a behavioural test, requirement to neuter, lead and muzzle in public. Must be walked by someone over 18, needs insurance, has to be a single pet in the household
	Thailand	Not illegal to own but cannot import into the country
	Ukraine	Listed as a dangerous dog breed
	United Arab Emirates	Prohibited - cannot be imported into the country. All pet dogs vaccinated, registered, on lead in public with large dogs muzzled
	United States	Regulations depend on the state and city

Sources

https://www.petraveller.com.au/blog/tag/banned-breeds
Bermuda Islands: https://www.gov.bm/regulations-pertaining-animals-island
Denmark: https://www.petraveller.com.au/blog/banned-dog-breeds-in-denmark
France: https://www.petraveller.com.au/blog/banned-dog-breeds-in-france
Iceland: https://www.pettravel.com/immigration/Iceland.cfm ;
https://www.icelandair.com/support/special-assistance/animal-transportation/
Ireland: https://www.petraveller.com.au/blog/banned-breeds-in-ireland
Malaysia: https://www.petraveller.com.au/blog/banned-and-restricted-dog-breeds-in-malaysia
Portugal: https://www.angloinfo.com/how-to/portugal/family/pets-animals/dangerous-dogs ;
https://www.dgav.pt/animais/conteudo/animais-de-companhia-2/bem-estar-animal/animais-perigosos-e-potencialmente-perigosos/treino-de-caes-perigosos-e-potencialmente-perigosos/
Singapore: https://www.petraveller.com.au/blog/banned-cat-and-dog-breeds-in-singapore
Spain: https://www.petraveller.com.au/blog/banned-breeds-in-spain
Switzerland: https://www.petraveller.com.au/blog/banned-breeds-in-switzerland
Thailand: https://www.petraveller.com.au/blog/banned-breeds-in-thailand
United Arab Emirates: https://www.petraveller.com.au/blog/banned-dog-breeds-in-the-united-arab-emirates
United States: https://bslcensus.com/

Supplementary Table S4. ANOVA results for the comparison of DIAS OQS and each factor between legislated and non-legislated groups, as a function of legislation status, neuter status, sex, and their interactions. Tables include unadjusted and Bonferroni adjusted *P* values, alongside partial eta squared (*ηp^2).*

Factor	*Sum. Sq.*	*df*	*F*	*P*	P_{adj}	ηp^2
**Model: OQS ~ Legislation*Neuter*Sex**						
Legislation	0.0792	1	6.73	0.0096	0.0670	0.0036
Neuter	0.0255	1	2.17	0.1409	0.9863	0.0012
Sex	0.0737	1	6.26	0.0125	0.0872	0.0034
Legislation:neuter	0.0023	1	0.2	0.6579	1	0.0001
Legislation:sex	0.0212	1	1.8	0.1797	1	0.0010
Neuter:sex	0.0059	1	0.5	0.479	1	0.0003
Legislation:neuter:sex	0.0001	1	0.01	0.941	1	0.0000
Residuals	21.6281	1837				
**Model: Behavioural Regulation ~ Legislation*Neuter*Sex**						
Legislation	0.123	1	4.60	0.0321	0.2243	0.0025
Neuter	0.047	1	1.78	0.1822	1	0.0010
Sex	0.099	1	3.72	0.0539	0.3774	0.0020
Legislation:neuter	0.001	1	0.04	0.8405	1	0.0000
Legislation:sex	0.023	1	0.87	0.3524	1	0.0005
Neuter:sex	0.044	1	1.66	0.1977	1	0.0009
Legislation:neuter:sex	0.002	1	0.06	0.8119	1	0.0000
Residuals	48.93	1837				
**Model: Aggression and Novelty ~ Legislation*Neuter*Sex**						
Legislation	0.005	1	0.21	0.6484	1	0.0001
Neuter	0.399	1	17.94	< 0.0001	0.0002	0.0097
Sex	0.026	1	1.17	0.2801	1	0.0006
Legislation:neuter	0.017	1	0.75	0.3867	1	0.0004
Legislation:sex	0.054	1	2.45	0.1180	0.8262	0.0013
Neuter:sex	0.044	1	1.98	0.1598	1	0.0011
Legislation:neuter:sex	0.059	1	2.65	0.1039	0.7274	0.0014
Residuals	40.846	1837				
**Model: Responsiveness ~ Legislation*Neuter*Sex**						
Legislation	0.0120	1	0.70	0.4022	1	0.0004
Neuter	0.2643	1	15.51	< 0.0001	0.0006	0.0084
Sex	0.0097	1	0.57	0.4501	1	0.0003
Legislation:neuter	0.0083	1	0.49	0.4841	1	0.0003
Legislation:sex	0.0050	1	0.29	0.5894	1	0.0002
Neuter:sex	0.0196	1	1.15	0.2834	1	0.0006
Legislation:neuter:sex	0.0053	1	0.31	0.5771	1	0.0002
Residuals	31.2920	1837				

Supplementary Table S5. Effect size estimates and confidence intervals of the difference between legislated and non-legislated groups for DIAS and PANAS variables. Mean differences and associated CIs were calculated via non-parametric bootstrapping. The CLES expresses effect size in terms of the probability that a randomly selected individual in one group will score higher than a randomly selected individual in the other group (McGraw & Wong, 1992). CLES for *Aggression Threshold and Response to Novelty* may be slightly biased due to negatively skewed distribution. Parametric confidence intervals were computed for Cohen D as provided within the lsr cohen.d function, and these did not differ substantially from non-parametric Bias and Accelerated (BCa) 95% confidence intervals computed using the *Boot* function, so the parametric form is reported here.

	**Mean difference [CI]**	**Cohen D [CI]**	**CLES**
DIAS Overall Questionnaire Score	0.01 [0.00, 0.03]	0.13 [0.03, 0.23]	0.54
DIAS Behavioural Regulation	0.02 [0.00, 0.03]	0.11 [0.01, 0.21]	0.53
DIAS Aggression Threshold & Response to Novelty	0.00 [-0.02, 0.01]	-0.03 [-1.14, 0.07]	0.49
DIAS Responsiveness	0.00 [-0.02, 0.01]	-0.04 [-0.14, 0.06]	0.49
PANAS Positive Activation	0.01 [0.00, 0.02]	0.08 [0.00, 0.18]	0.52
PANAS Negative Activation	0.00 [-0.02, 0.01]	-0.03 [0.07, -0.13]	0.49

Supplementary Table S6. ANOVA results for the comparison of DIAS OQS and each factor between breeds as a function of breed, neuter status, and sex. Tables include unadjusted and Bonferroni adjusted *P* values, alongside partial eta squared (ηp^2).

Factor	***Sum. Sq.***	***df***	***F***	***P***	P_{adj}	ηp^2
**Model: OQS ~ Breed+Neuter+Sex**						
Breed	0.8900	24	3.22	< 0.0001	< 0.0001	0.0493
Neuter	0.0272	1	3.36	0.1248	0.3745	0.0016
Sex	0.0278	1	2.41	0.1205	0.3614	0.0034
Residuals	17.1506	1487				
**Model: Behavioural Regulation ~ Breed+Neuter+Sex**						
Breed	1.909	24	3.00	< 0.0001	< 0.0001	0.0461
Neuter	0.055	1	2.08	0.1495	0.4484	0.0014
Sex	0.035	1	1.31	0.2534	0.7601	0.0009
Residuals	39.467	1487				
**Model: Aggression and Novelty ~ Breed+Neuter+Sex**						
Breed	2.8577	24	5.65	<0.0001	< 0.0001	0.0835
Neuter	0.3016	1	14.31	< 0.0001	0.0005	0.0095
Sex	0.0096	1	0.45	0.50010	1	0.0003
Residuals	31.3492	1487				
**Model: Responsiveness ~ Breed+Neuter+Sex**						
Breed	2.8528	24	8.11	< 0.0001	< 0.0001	0.1157
Neuter	0.1536	1	10.47	0.0012	0.0037	0.0070
Sex	0.0276	1	1.88	0.1704	0.5113	0.0013
Residuals	21.7998	1487				

Supplementary Table S7. ANOVA results for the comparison of the two PANAS factors between legislated and non-legislated groups as a function of legislation status, neuter status, sex, and their interactions. Tables include unadjusted and Bonferroni-adjusted *P* values, alongside partial eta squared (ηp^2).

Factor	***Sum. Sq.***	***df***	***F***	***P***	P_{adj}	ηp^2
**Model: Positive Activation ~ Legislation*Neuter*Sex**						
Legislation	0.0327	1	1.99	0.1581	1	0.0011
Neuter	0.5821	1	35.47	< 0.0001	< 0.0001	0.0190
Sex	0.0140	1	0.85	0.3563	1	0.0005
Legislation:neuter	0.0263	1	1.60	0.2054	1	0.0009
Legislation:sex	0.0008	1	0.05	0.8285	1	0.0000
Neuter:sex	0.0000	1	0.00	0.9562	1	0.0000
Legislation:neuter:sex	0.0003	1	0.02	0.8972	1	0.0000
Residuals	30.6426	1837				
**Model: Negative Activation ~ Legislation*Neuter*Sex**						
Legislation	0.003	1	0.12	0.7324	1	0.0001
Neuter	1.171	1	48.92	< 0.0001	< 0.0001	0.0255
Sex	0.033	1	1.36	0.2439	1	0.0007
Legislation:neuter	0.053	1	2.20	0.1383	0.9678	0.0012
Legislation:sex	0.002	1	0.06	0.8001	1	0.0000
Neuter:sex	0.063	1	2.65	0.1037	0.7257	0.0014
Legislation:neuter:sex	0.015	1	0.64	0.4224	1	0.0003
Residuals	44.671	1837				

Supplementary Table S8. ANOVA results for the comparison of the two PANAS factors between breeds as a function of breed, neuter status, and sex. Tables include unadjusted and Bonferroni-adjusted *P* values, alongside partial eta squared (ηp^2).

Factor	***Sum. Sq.***	***df***	***F***	***P***	P_{adj}	ηp^2
**Model: Positive Activation ~ Breed+Neuter+Sex**						
Breed	3.7564	24	11.36	< 0.0001	< 0.0001	0.1530
Neuter	0.2100	1	15.24	< 0.0001	0.0003	0.001
Sex	0.0172	1	1.25	0.2638	0.7915	0.0008
Residuals	20.7960	1509				
**Model: Negative Activation ~ Breed+Neuter+Sex**						
Breed	1.621	24	2.86	< 0.0001	< 0.0001	0.0435
Neuter	0.760	1	32.14	< 0.0001	< 0.0001	0.0209
Sex	0.022	1	0.92	0.337	1	0.0006
Residuals	35.683	1509				

Supplementary Figures

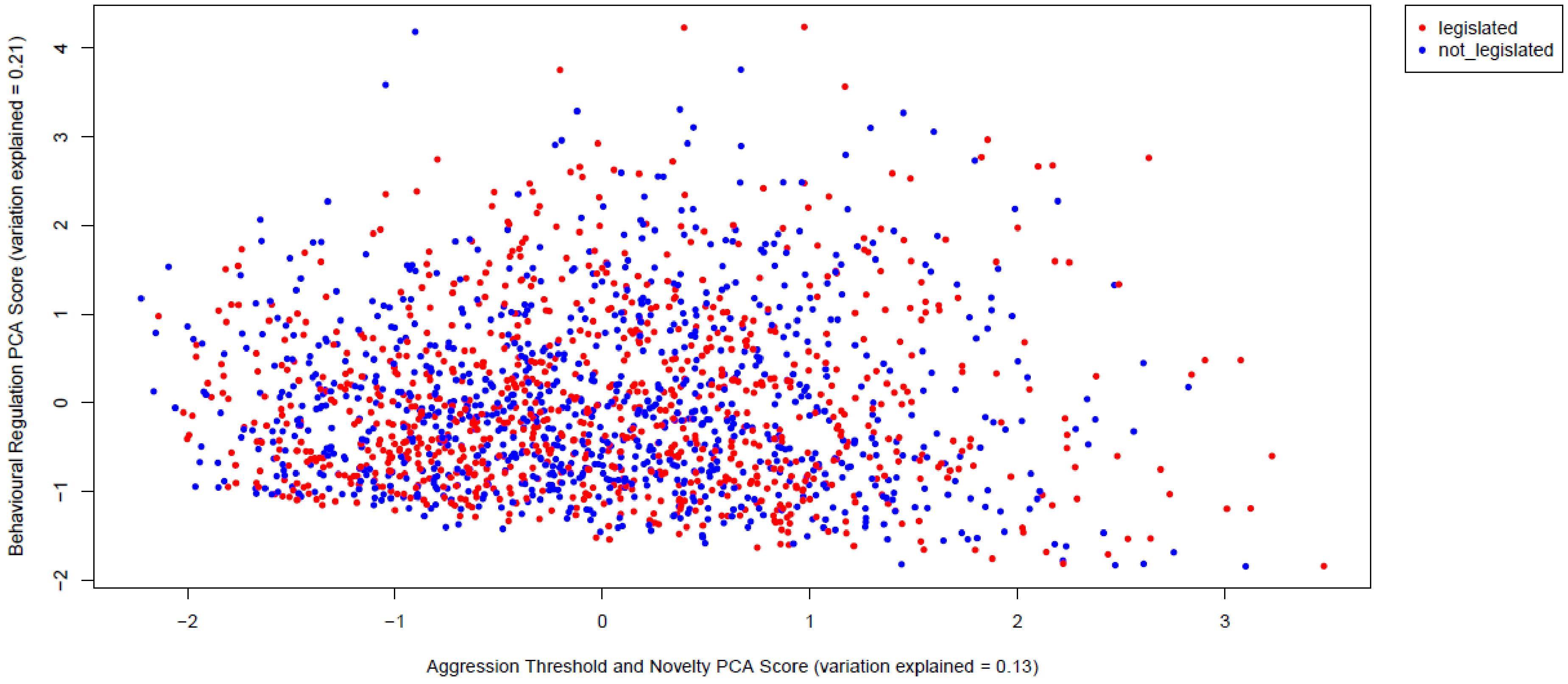

Supplementary Figure S1. PCA plot showing distribution of scores for two DIAS factors, *Behavioural Regulation* and *Aggression Threshold & Response to Novelty*, in legislated and non-legislated breed groups.

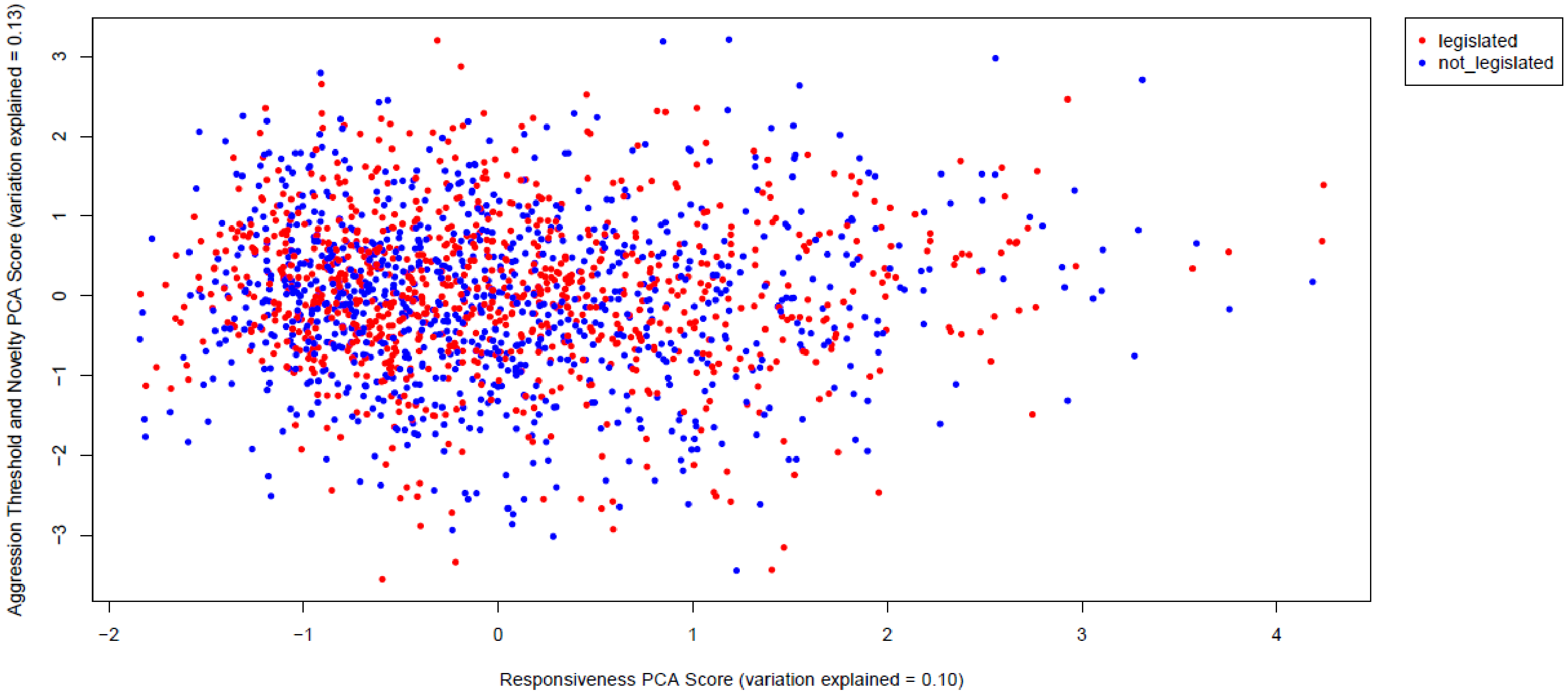

Supplementary Figure S2. PCA plot showing distribution of scores for two DIAS factors, *Aggression Threshold & Response to Novelty* and *Responsiveness*, in legislated and non-legislated breed groups.

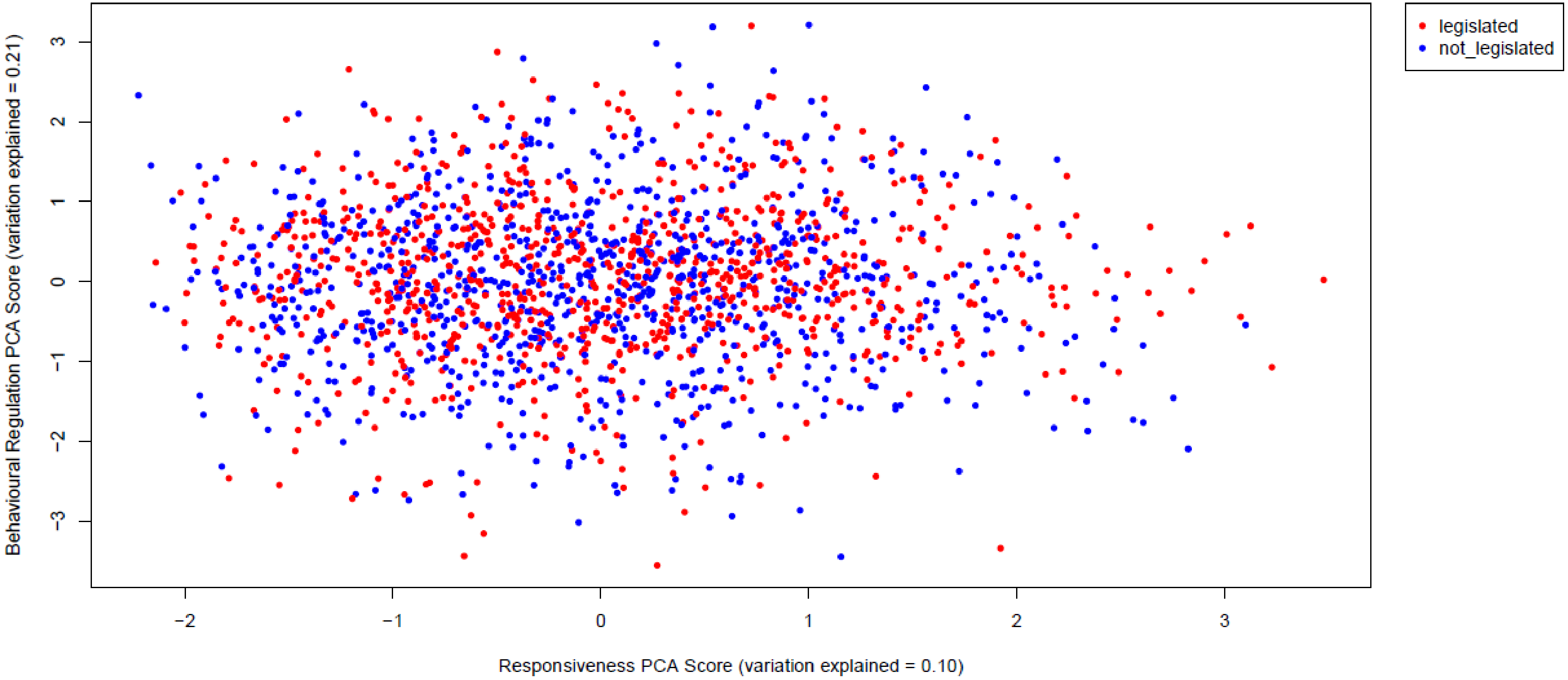

Supplementary Figure S3. PCA plot showing distribution of scores for two DIAS factors, *Responsiveness* and *Behavioural Regulation*, in legislated and non-legislated breed groups.

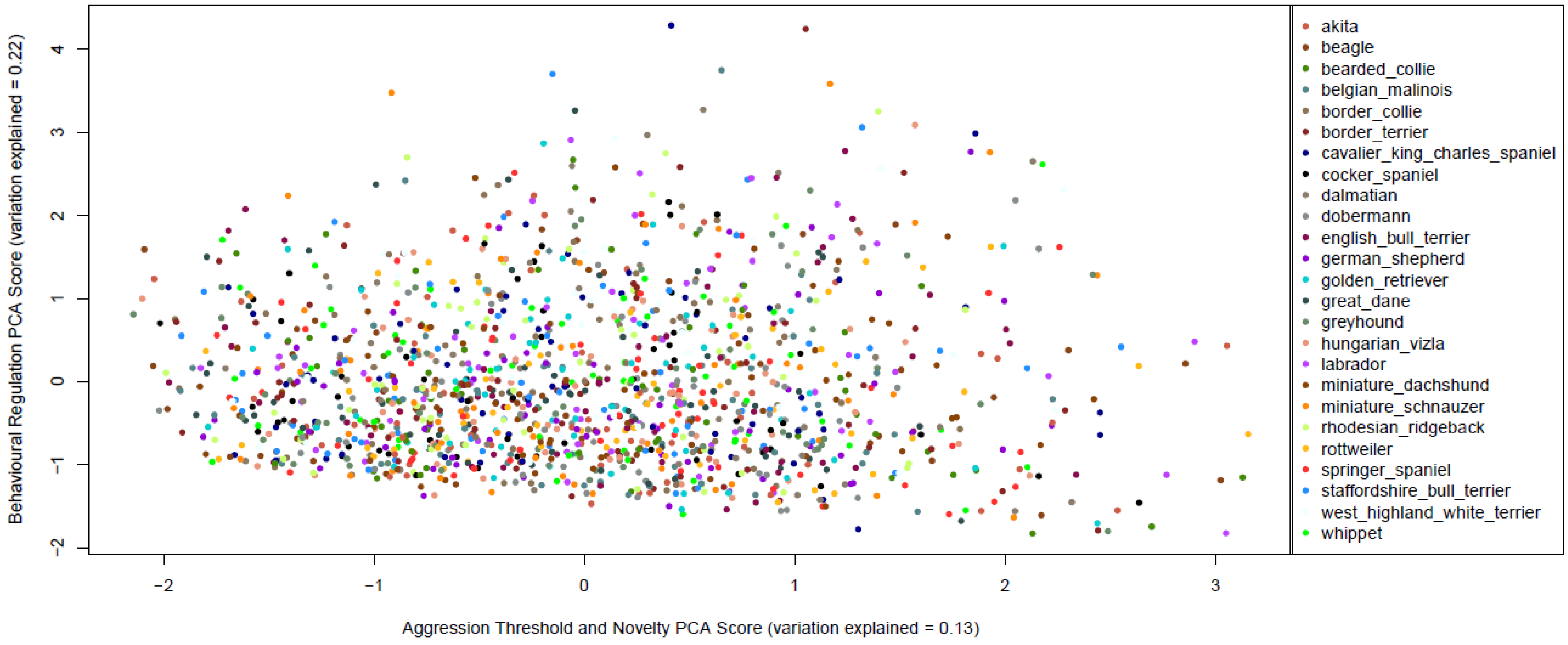

Supplementary Figure S4. PCA plot showing distribution of scores for two DIAS factors, *Behavioural Regulation* and *Aggression Threshold & Response to Novelty*, in dog breeds studied.

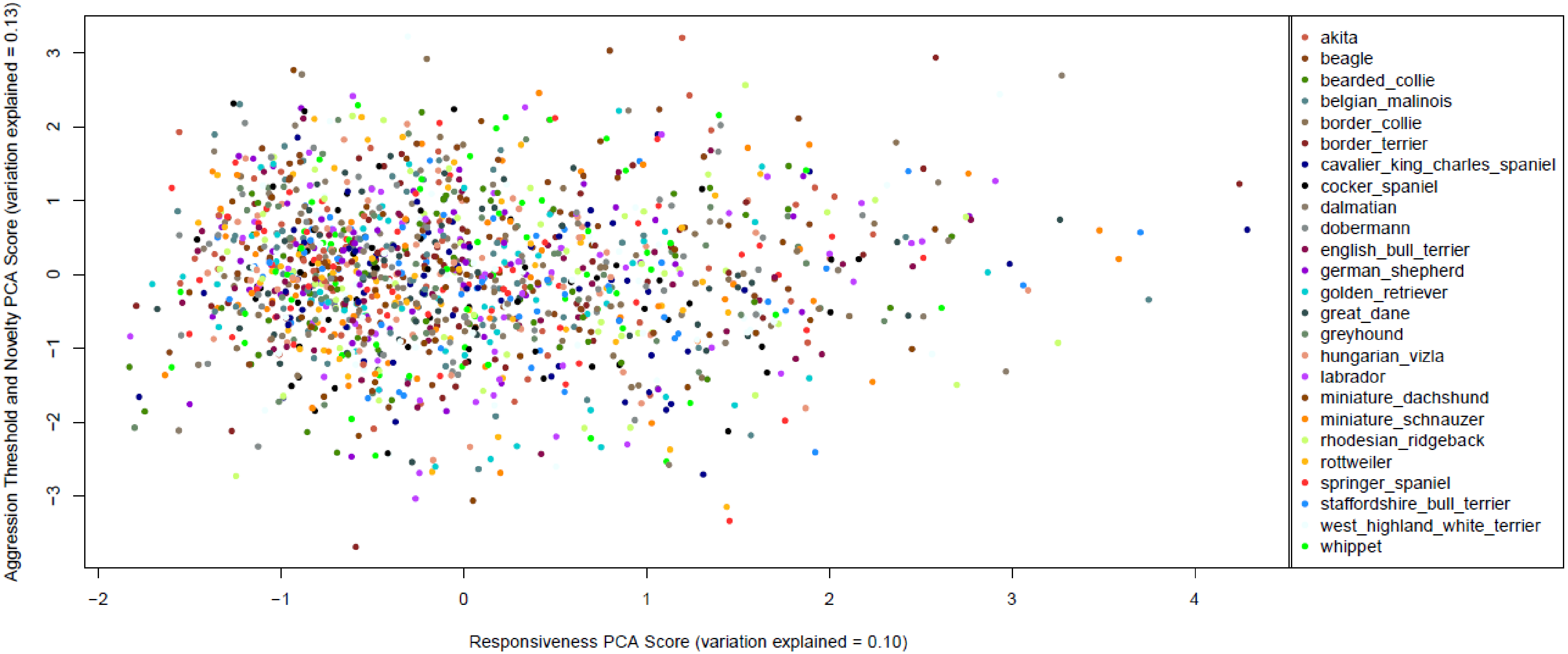

Supplementary Figure S5. PCA plot showing distribution of scores for two DIAS factors, *Aggression Threshold & Response to Novelty* and *Responsiveness*, in dog breeds studied.

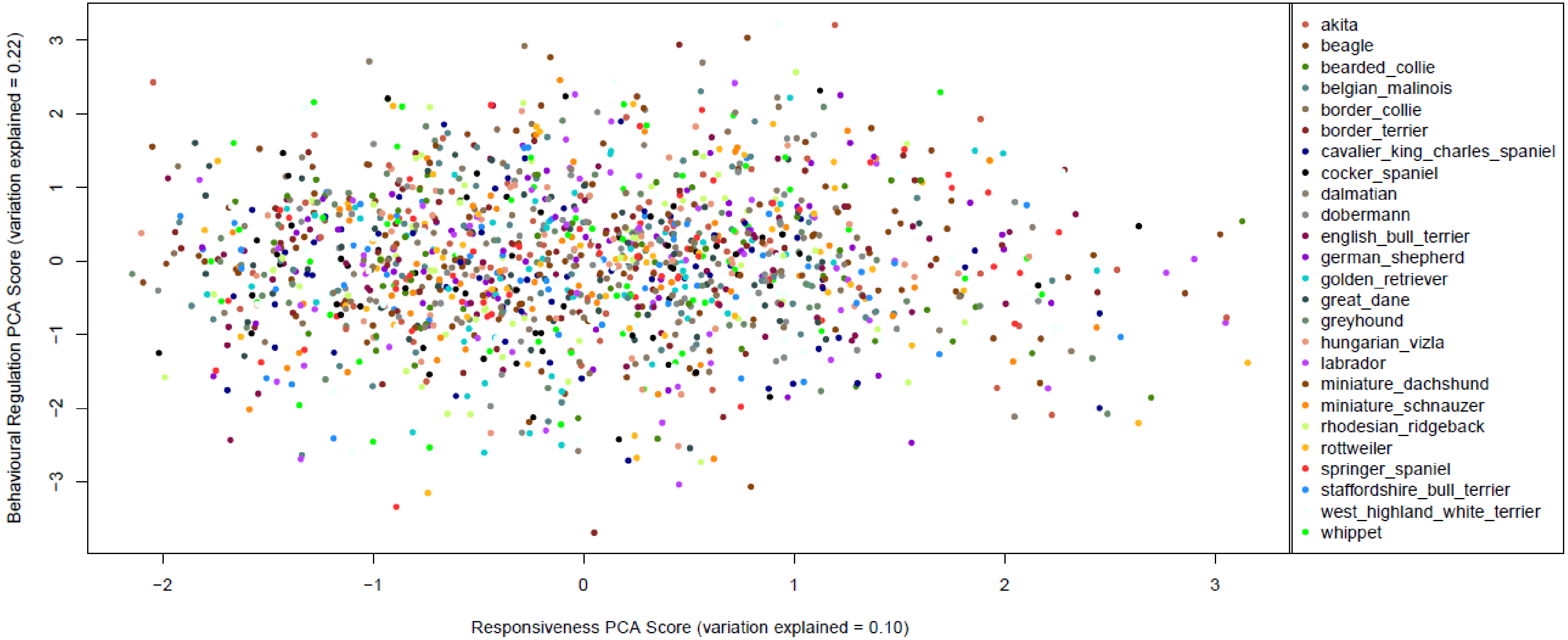

Supplementary Figure S6. PCA plot showing distribution of scores for two DIAS factors, *Responsiveness* and *Behavioural Regulation*, in dog breeds studied.

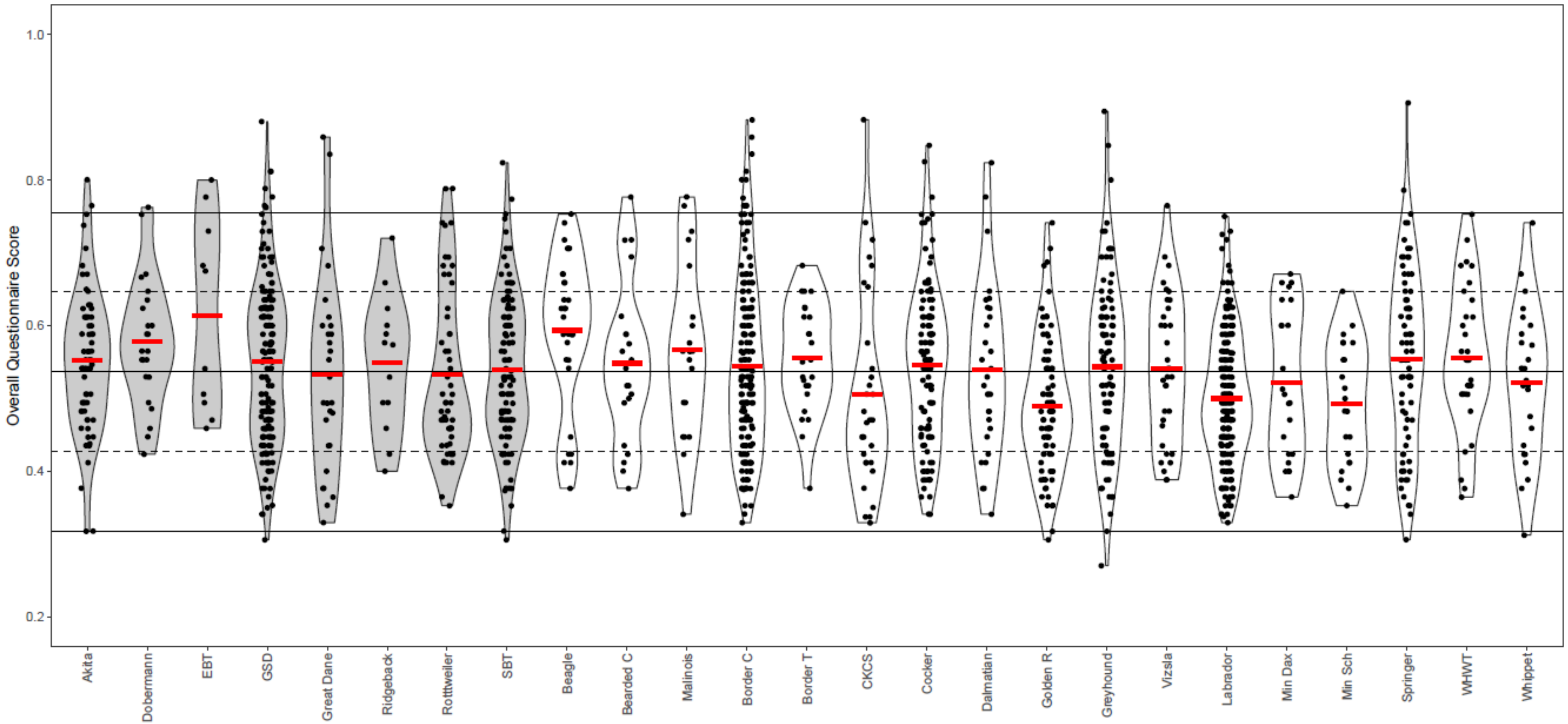

Supplementary Figure S7 A. Violin plot of DIAS Overall Questionnaire Score in the 25 breeds used in the breed level analysis. Each black dot within the box is an individual's score. The solid horizontal red line through each box represents the respective breed mean. The solid horizontal line through the entire plot represents the total sample mean. The upper and lower dotted line represents +/-1 SD from the mean, and the upper solid line represents +/- 2 SD from the mean.

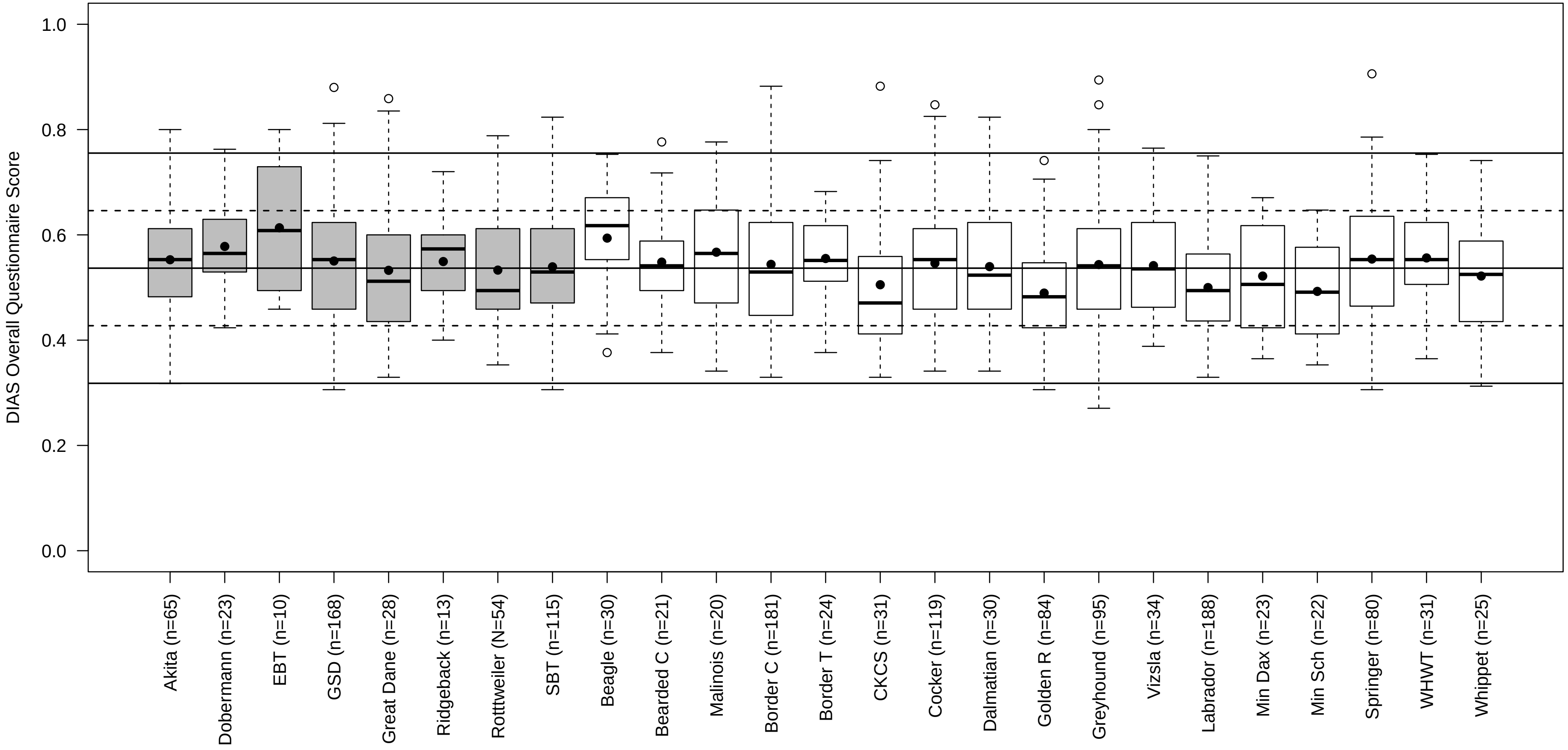

Supplementary Figure S7 B. Boxplot of DIAS Overall Questionnaire Score in the 25 breeds used in the breed level analysis. Solid points on each box represents the respective breed means. The solid horizontal line through plot represents the whole sample mean, horizontal dotted lines represent ± 1SD, and the upper & lower horizontal solid lines represent ± 2SD.

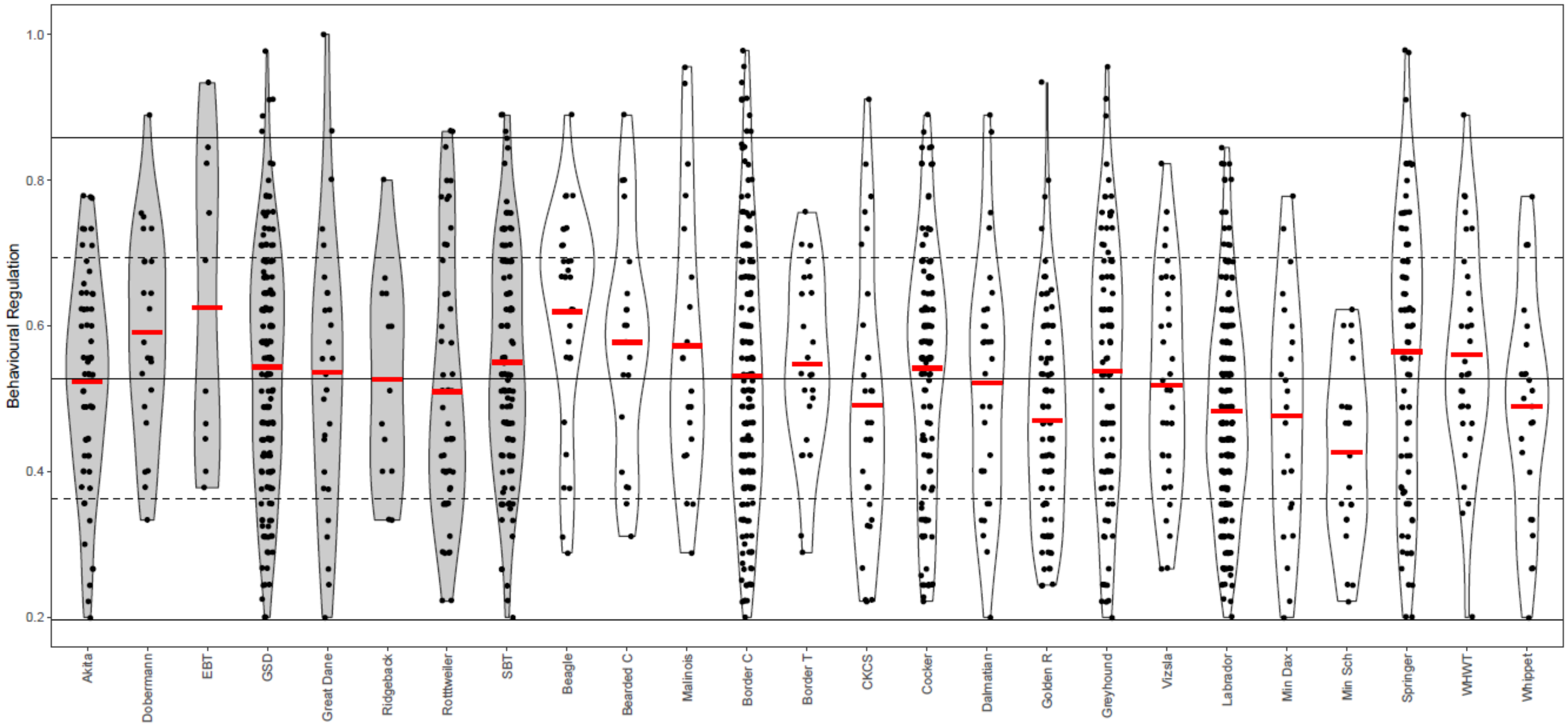

Supplementary Figure S8 A. Violin plot of DIAS Behavioural Regulation in the 25 breeds used in the breed level analysis. Each black dot within the box is an individual's score. The solid horizontal red line through each box represents the respective breed mean. The solid horizontal line through the entire plot represents the total sample mean. The upper and lower dotted line represents +/-1 SD from the mean, and the upper solid line represents +/- 2 SD from the mean.

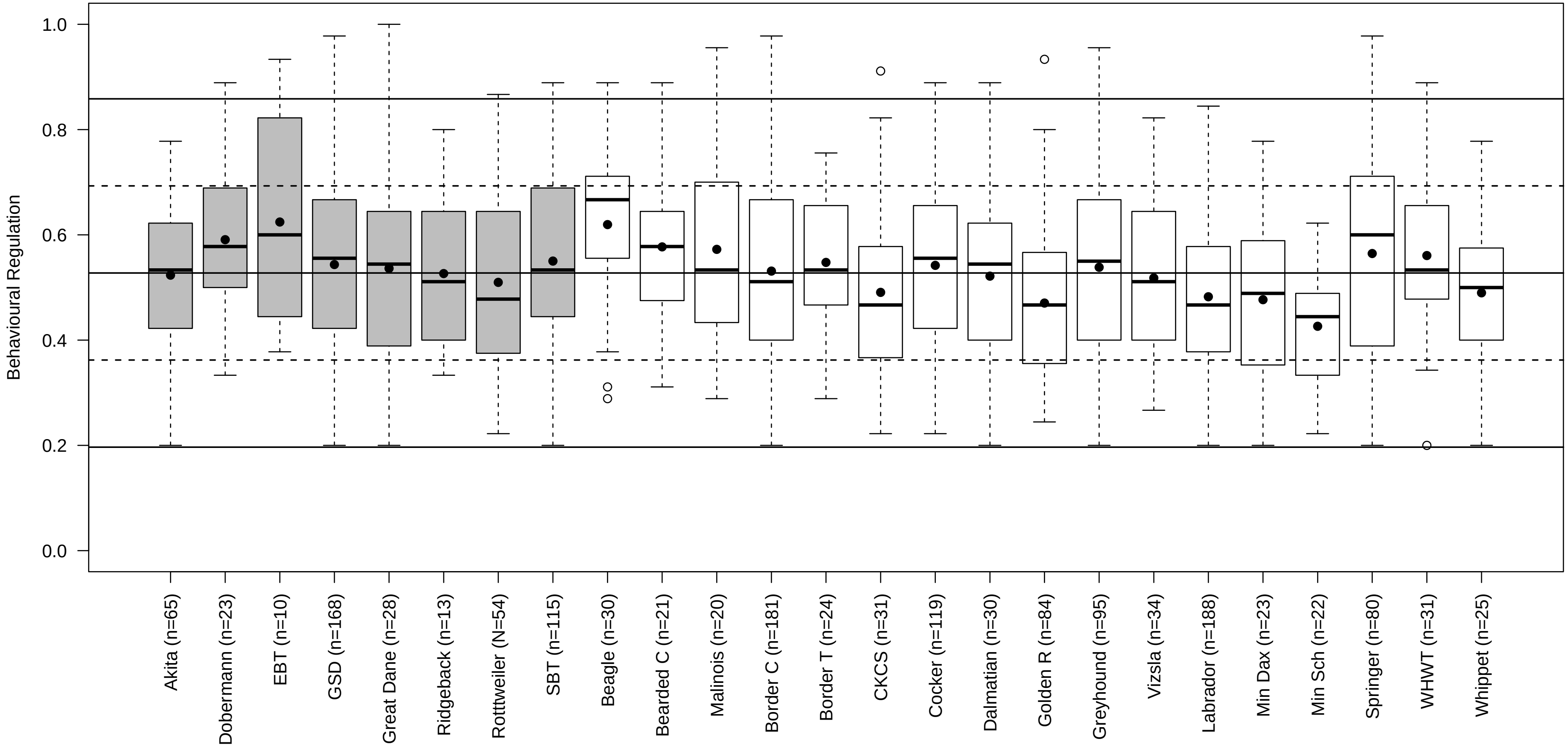

Supplementary Figure S8 B. Boxplot of DIAS Behavioural Regulation in the 25 breeds used in the breed level analysis. Solid points on each box represents the respective breed means. Solid horizontal line through plot represents the whole sample mean, horizontal dotted lines represent ± 1SD, and the upper & lower horizontal solid lines represent ± 2SD.

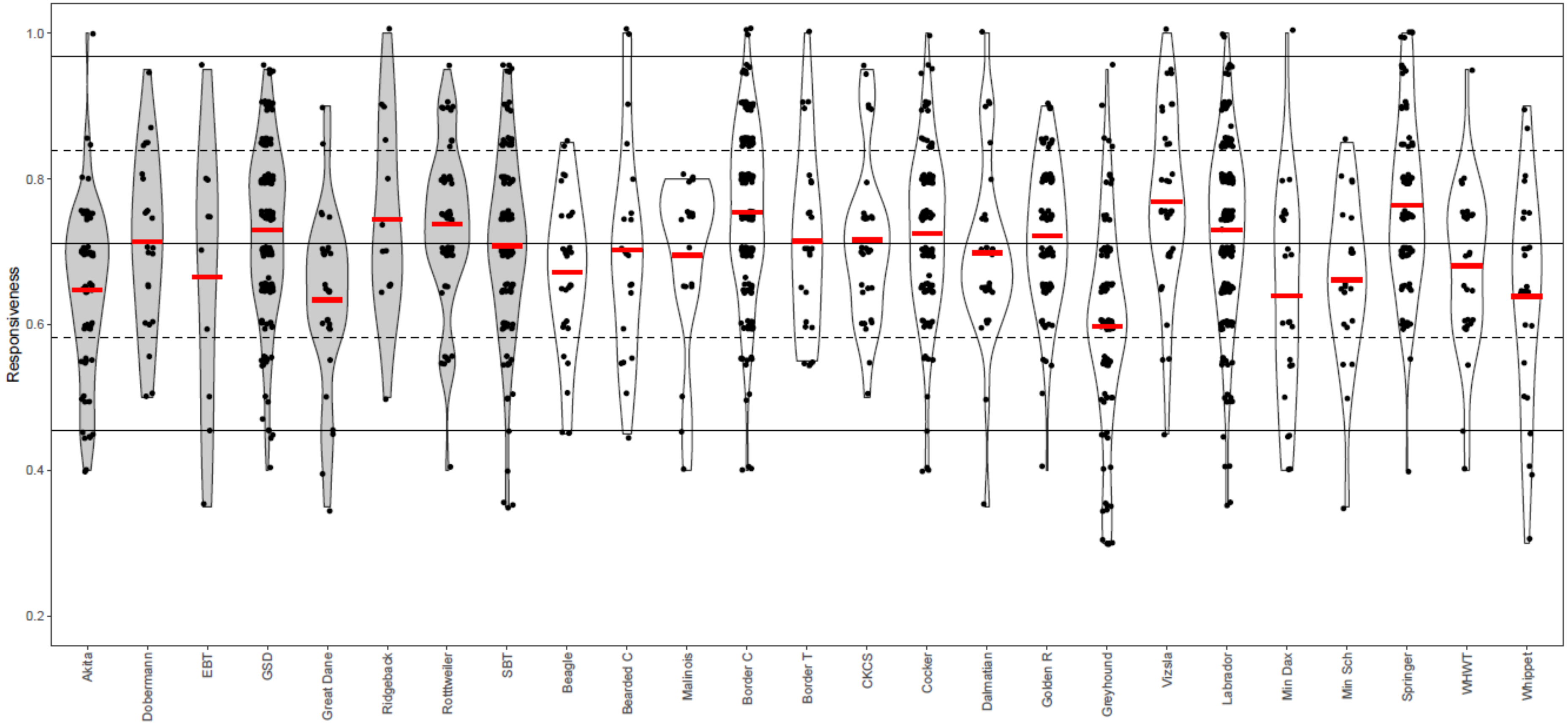

Supplementary Figure S9 A. Violin plot of DIAS Responsiveness in the 25 breeds used in the breed level analysis. Each black dot within the box is an individual's score. The solid horizontal red line through each box represents the respective breed mean. The solid horizontal line through the entire plot represents the total sample mean. The upper and lower dotted line represents +/-1 SD from the mean, and the upper solid line represents +/- 2 SD from the mean.

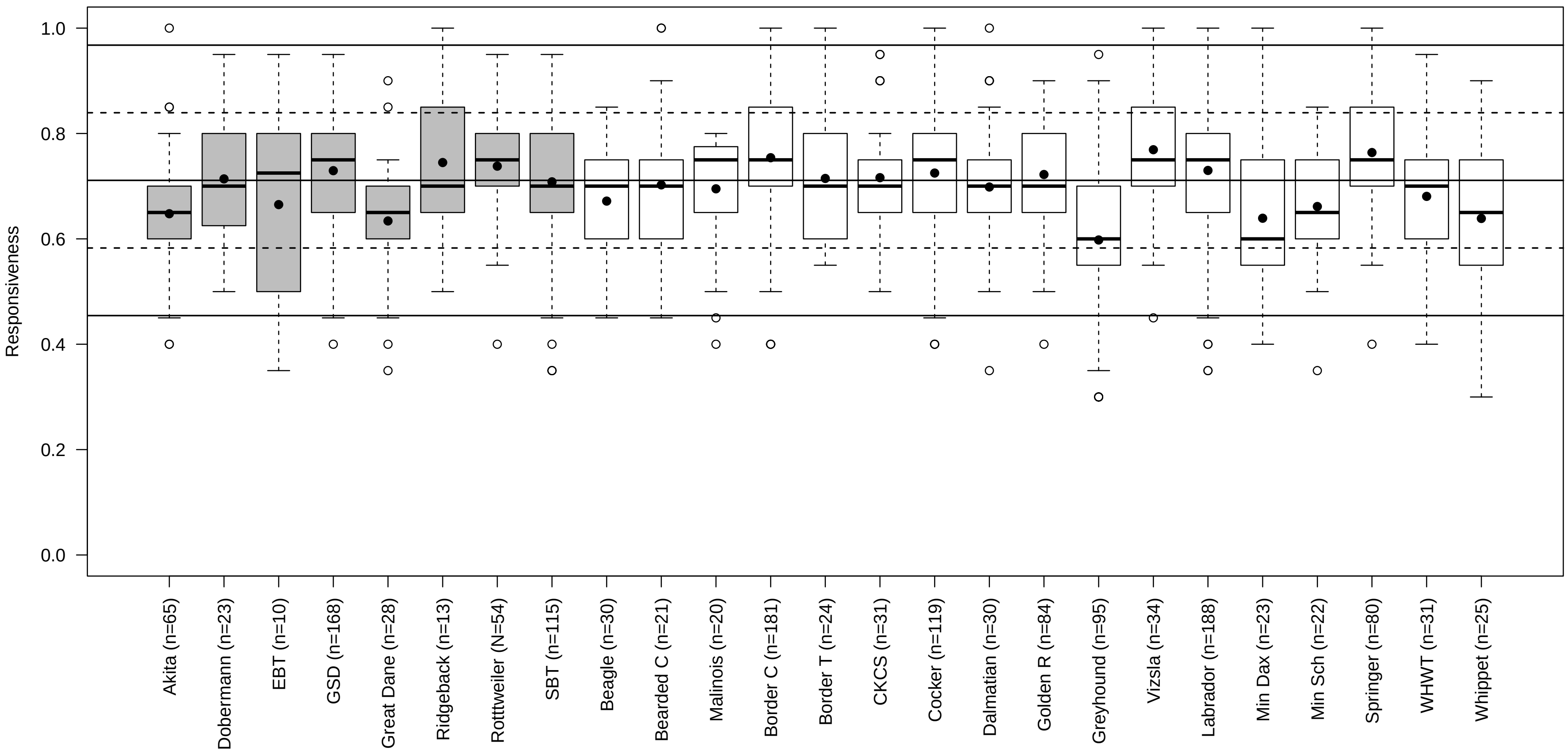

Supplementary Figure S9 B. Boxplot of DIAS Responsiveness in the 25 breeds used in the breed level analysis. Solid points on each box represents the respective breed means. Solid horizontal line through plot represents the whole sample mean, horizontal dotted lines represent ± 1SD, and the upper & lower horizontal solid lines represent ± 2SD.

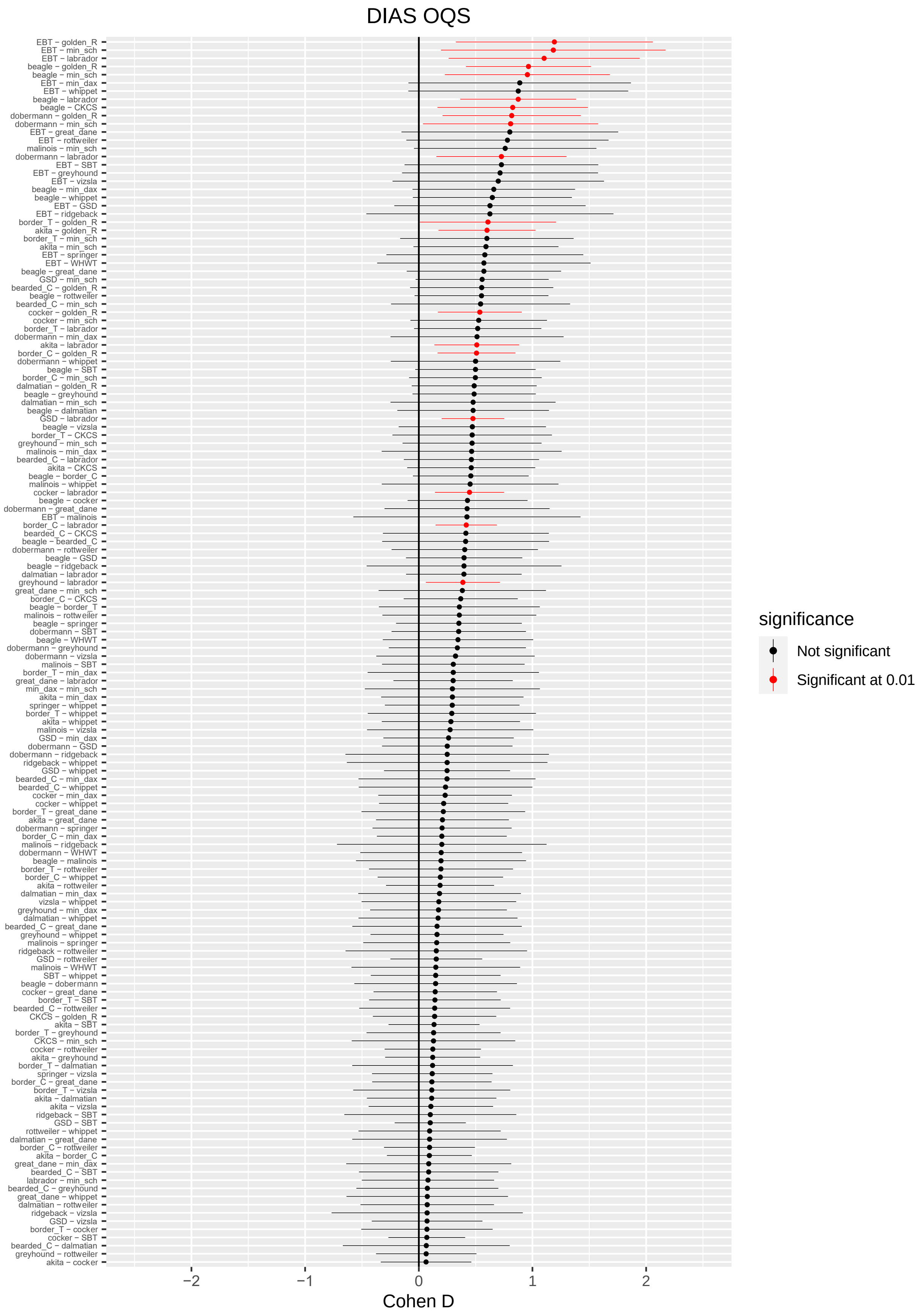

Supplementary Figure S10. Pairwise breed comparisons in DIAS Overall Questionnaire Score. Cohen D point estimates are shown with 99% Confidence Intervals. Statistical significance shown refers to pairwise t-tests using the pooled standard deviation.

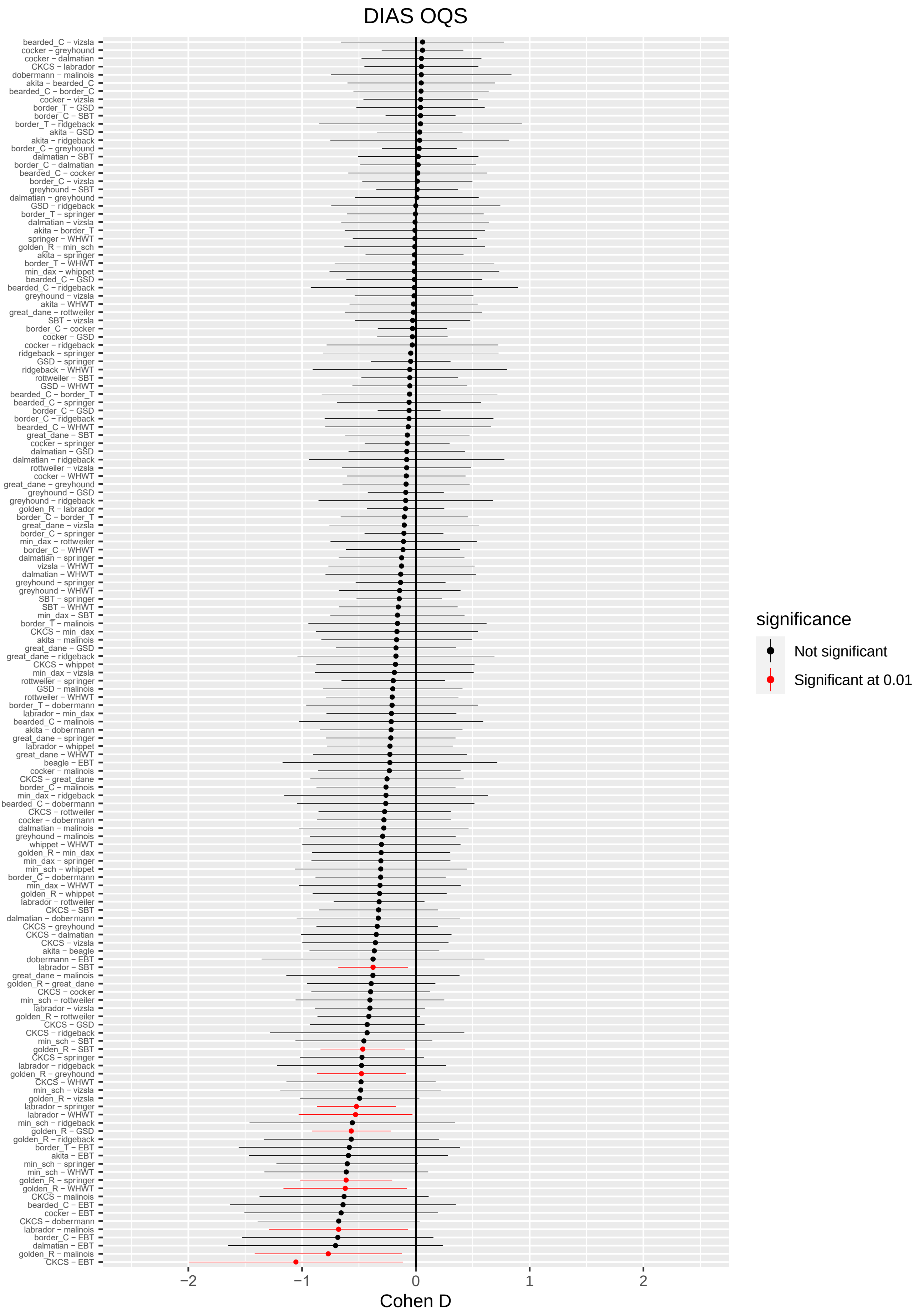

Supplementary Figure S10 continued.

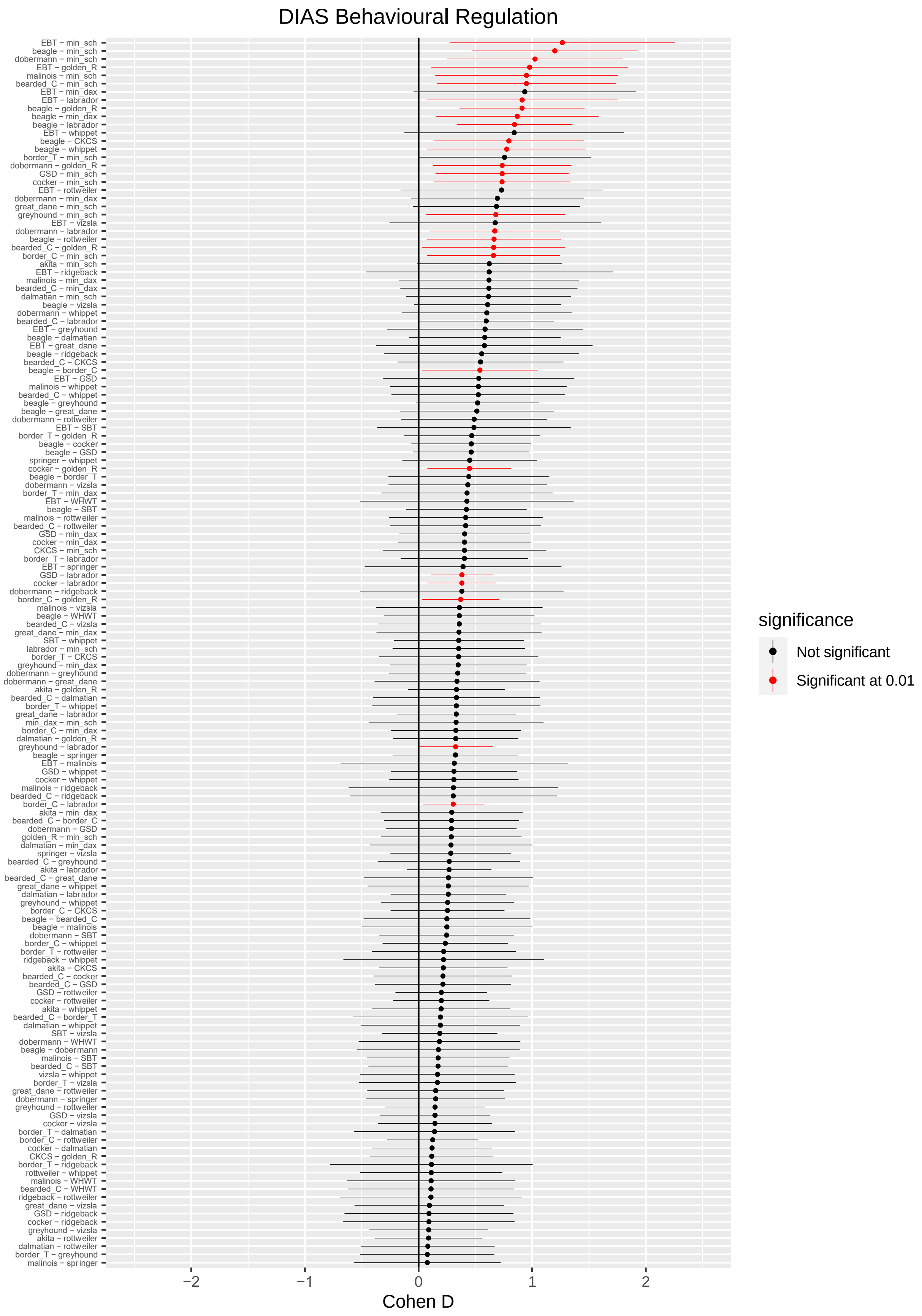

Supplementary Figure S11. Pairwise breed comparisons in DIAS Behavioural Regulation. Cohen D point estimates are shown with 99% Confidence Intervals. Statistical significance shown refers to pairwise t-tests using the pooled standard deviation.

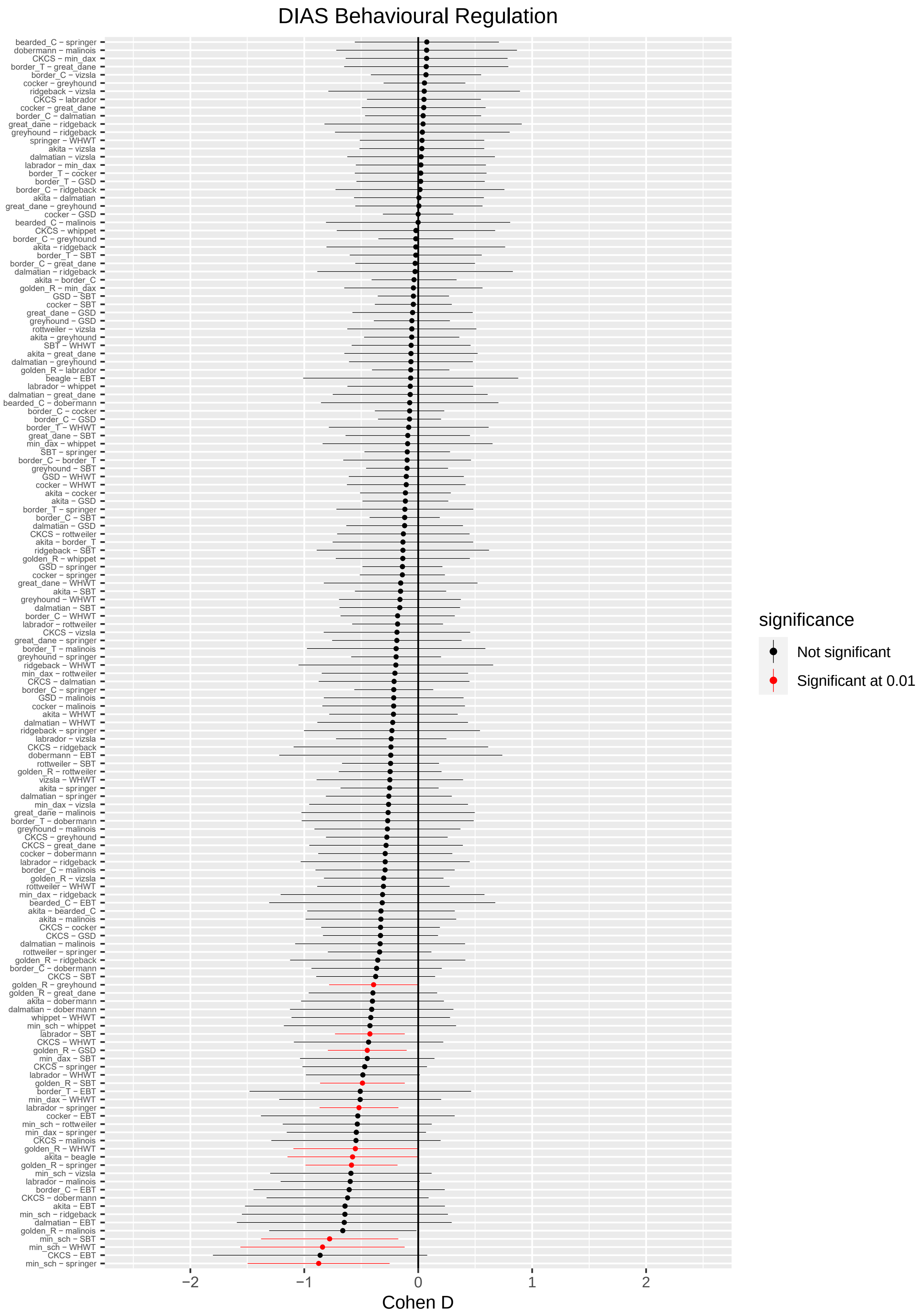

Supplementary Figure S11 continued.

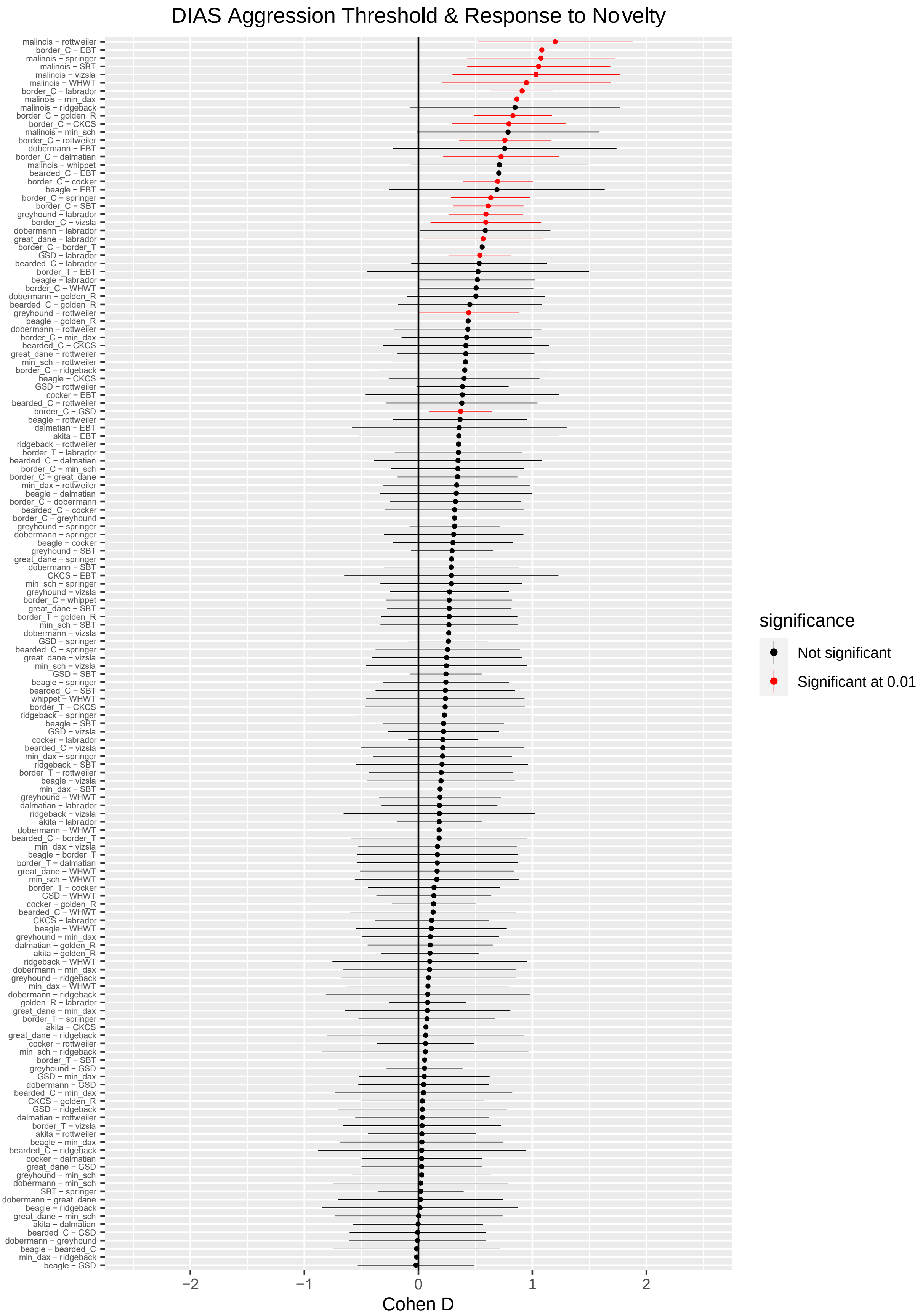

Supplementary Figure S12. Pairwise breed comparisons in DIAS Aggression Threshold and Response to Novelty. Cohen D point estimates are shown with 99% Confidence Intervals. Statistical significance shown refers to pairwise t-tests using the pooled standard deviation.

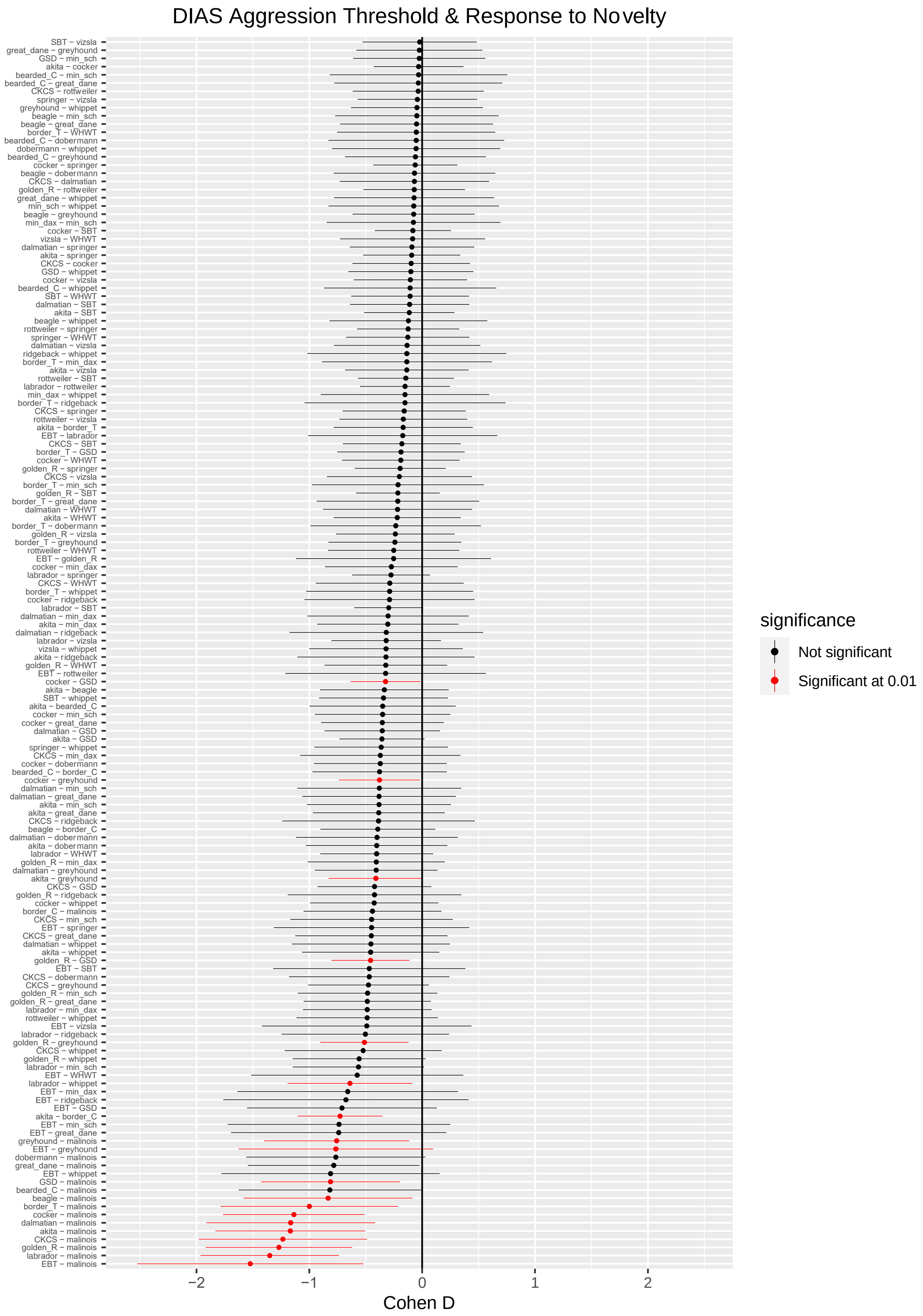

Supplementary Figure S12 continued.

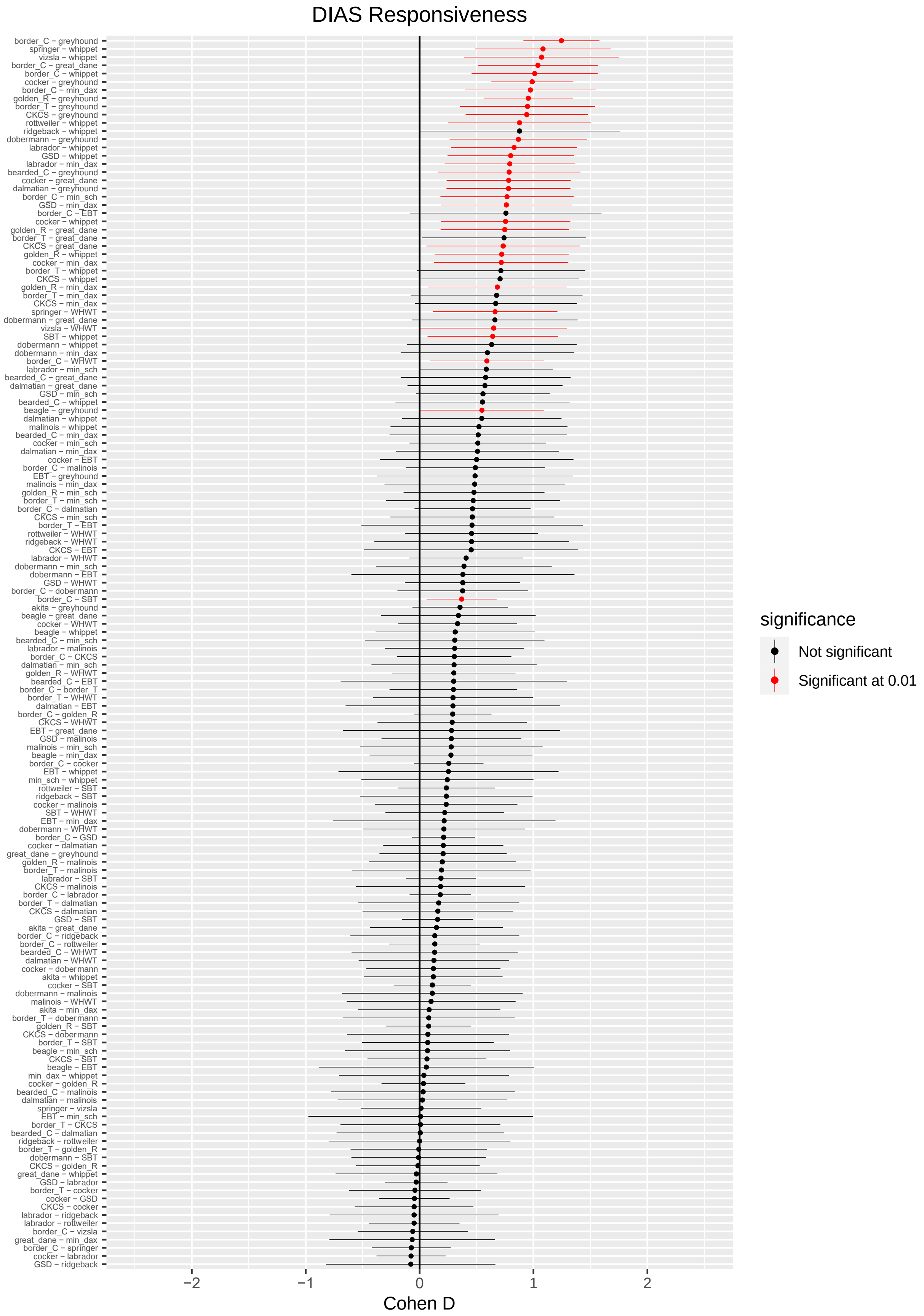

Supplementary Figure S13. Pairwise breed comparisons in DIAS Responsiveness. Cohen D point estimates are shown with 99% Confidence Intervals. Statistical significance shown refers to pairwise t-tests using the pooled standard deviation.

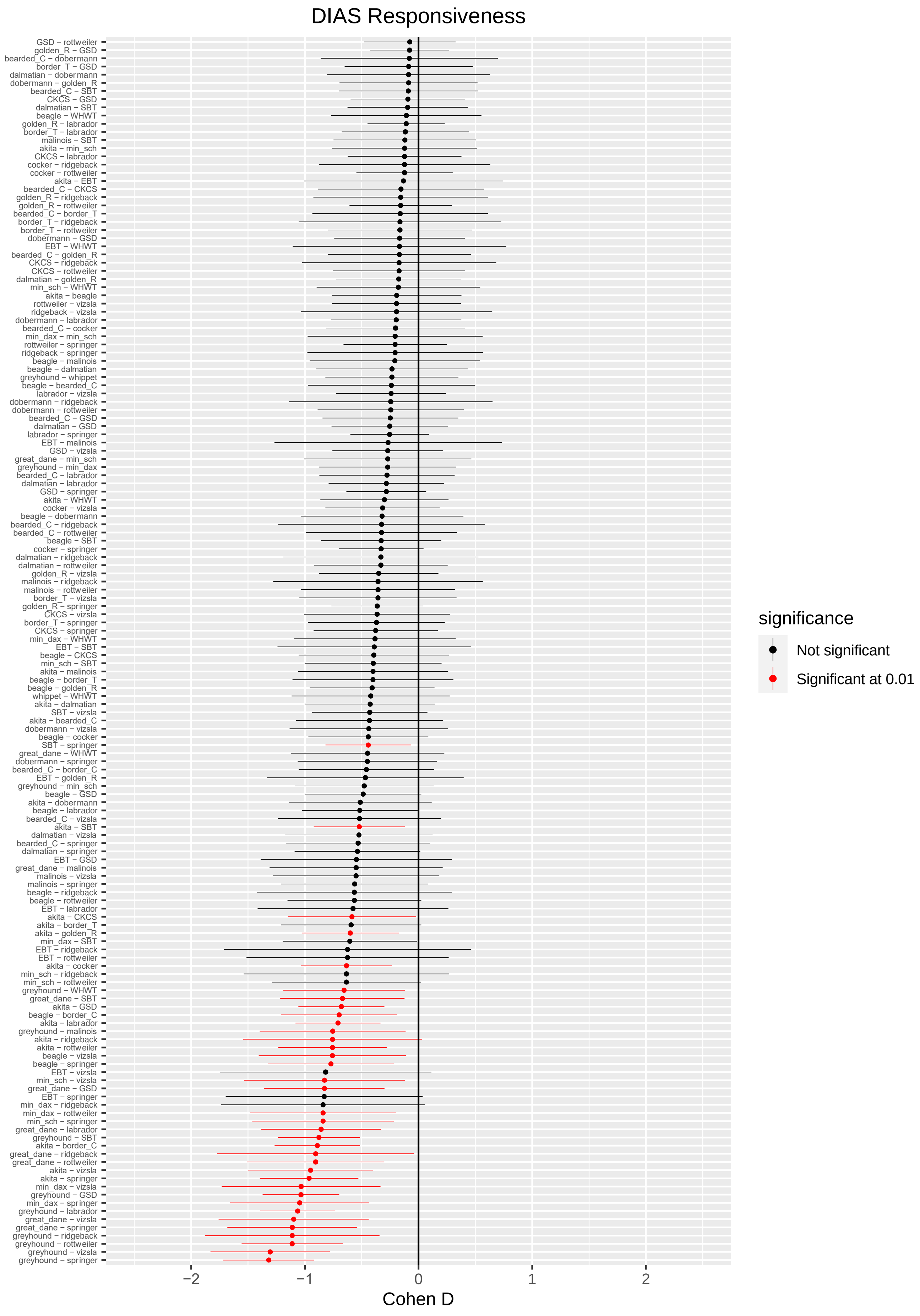

Supplementary Figure S13 continued.

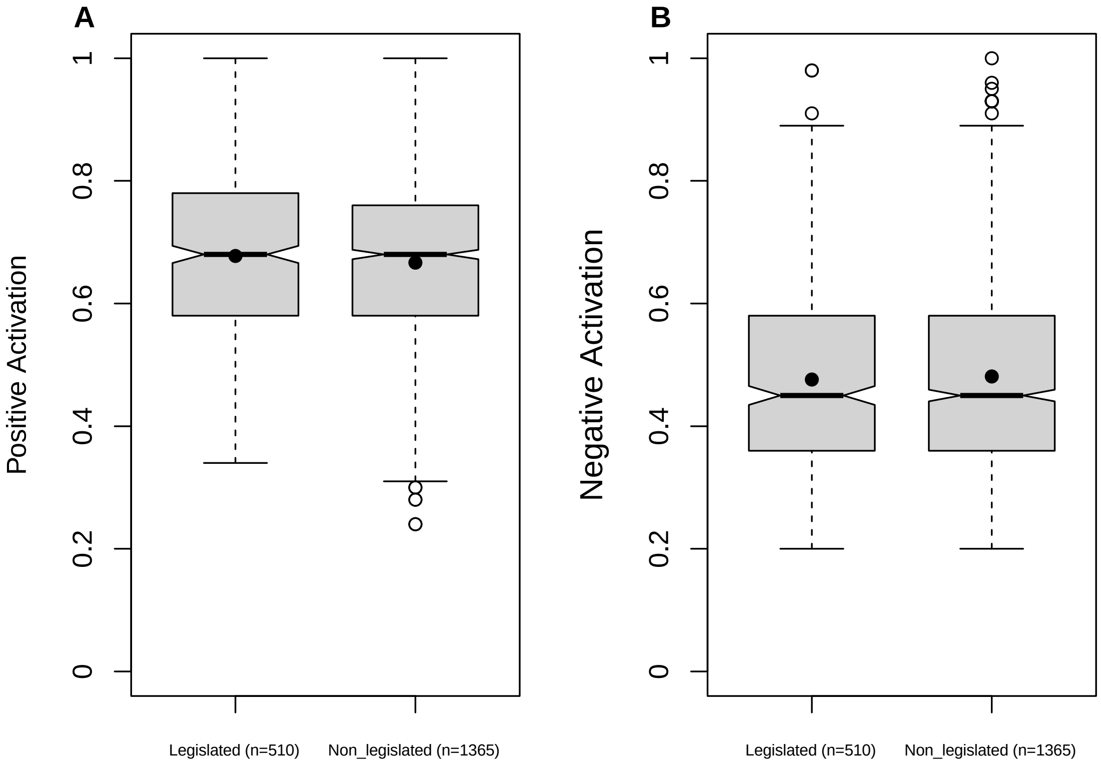

Supplementary Figure S14. Boxplots from PANAS questionnaire of A) positive activation, and B) negative activation, between legislation status groups.

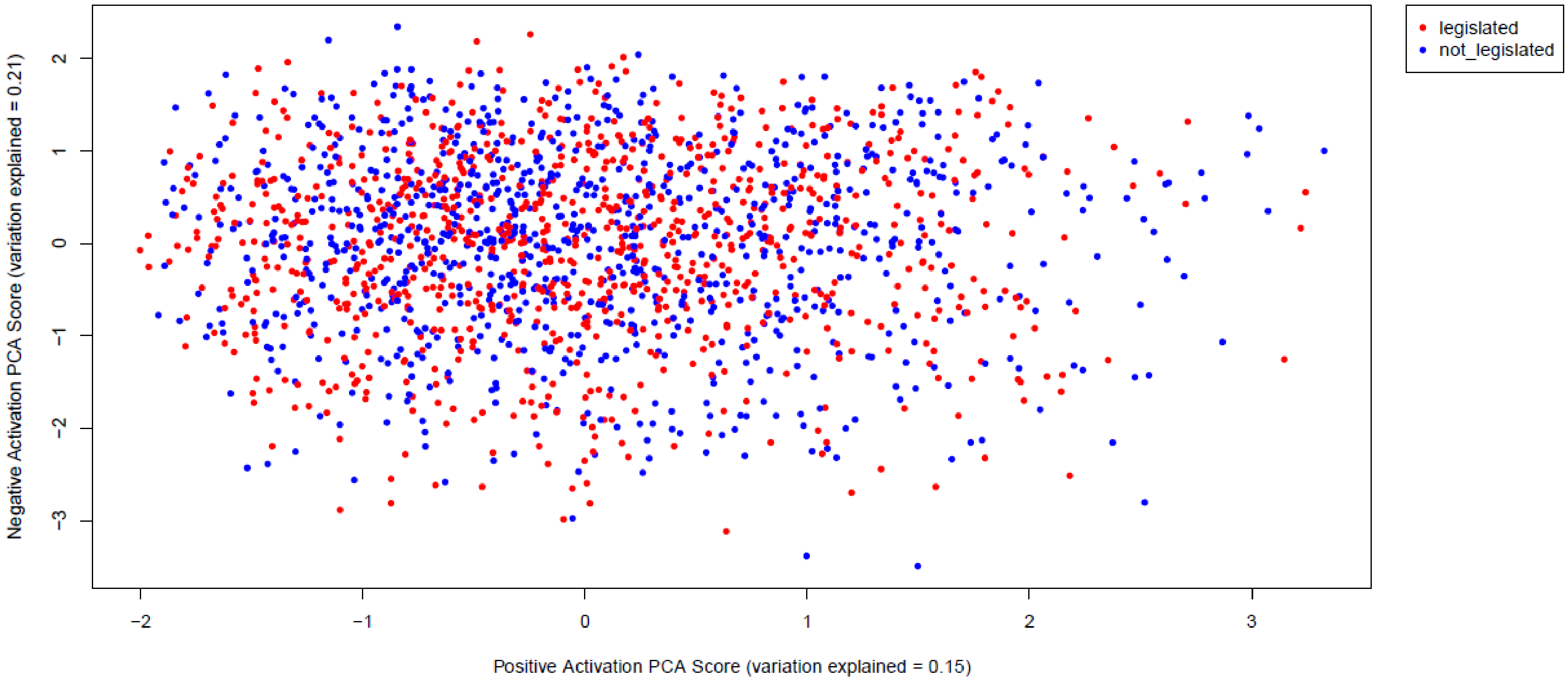

Supplementary Figure S15. PCA plot showing distribution of scores for two PANAS factors, *Positive* and *Negative Activation*, in legislated and non-legislated breed groups.

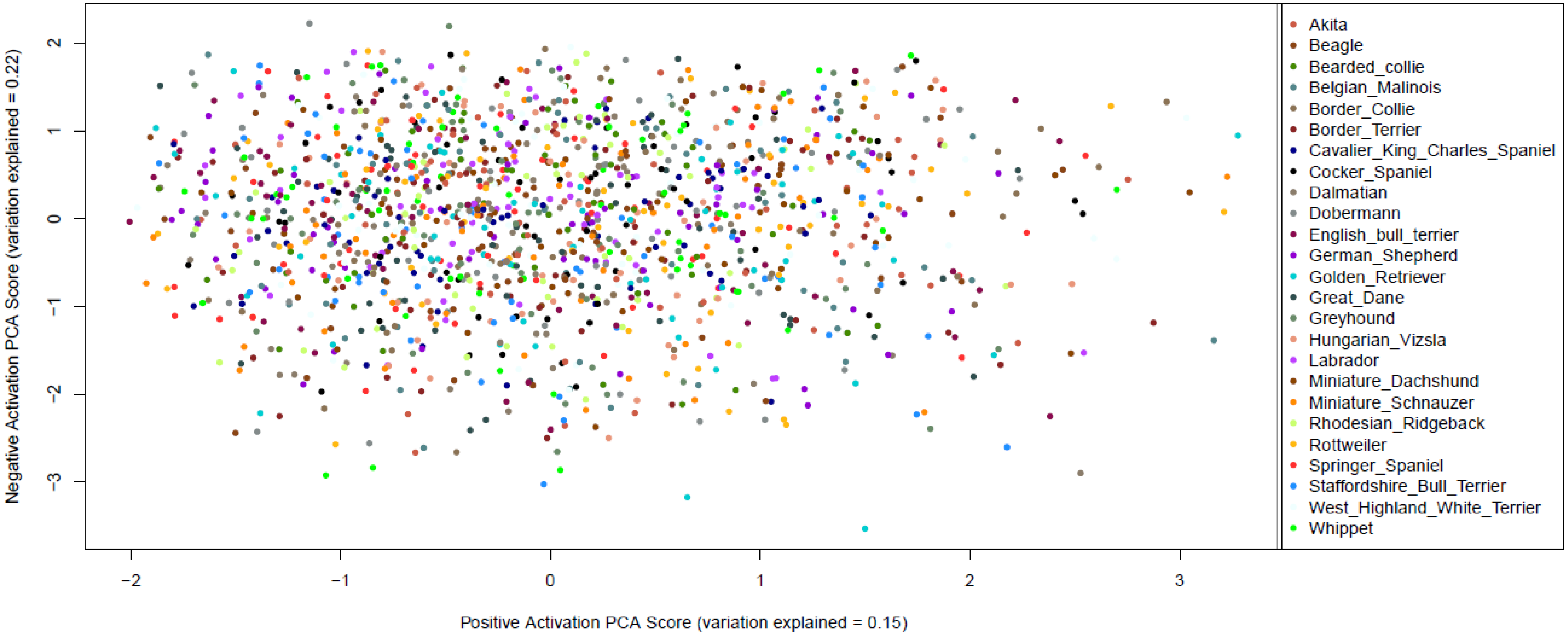

Supplementary Figure S16. PCA plot showing distribution of scores for two PANAS factors, *Positive* and *Negative Activation*, in dog breeds studied.

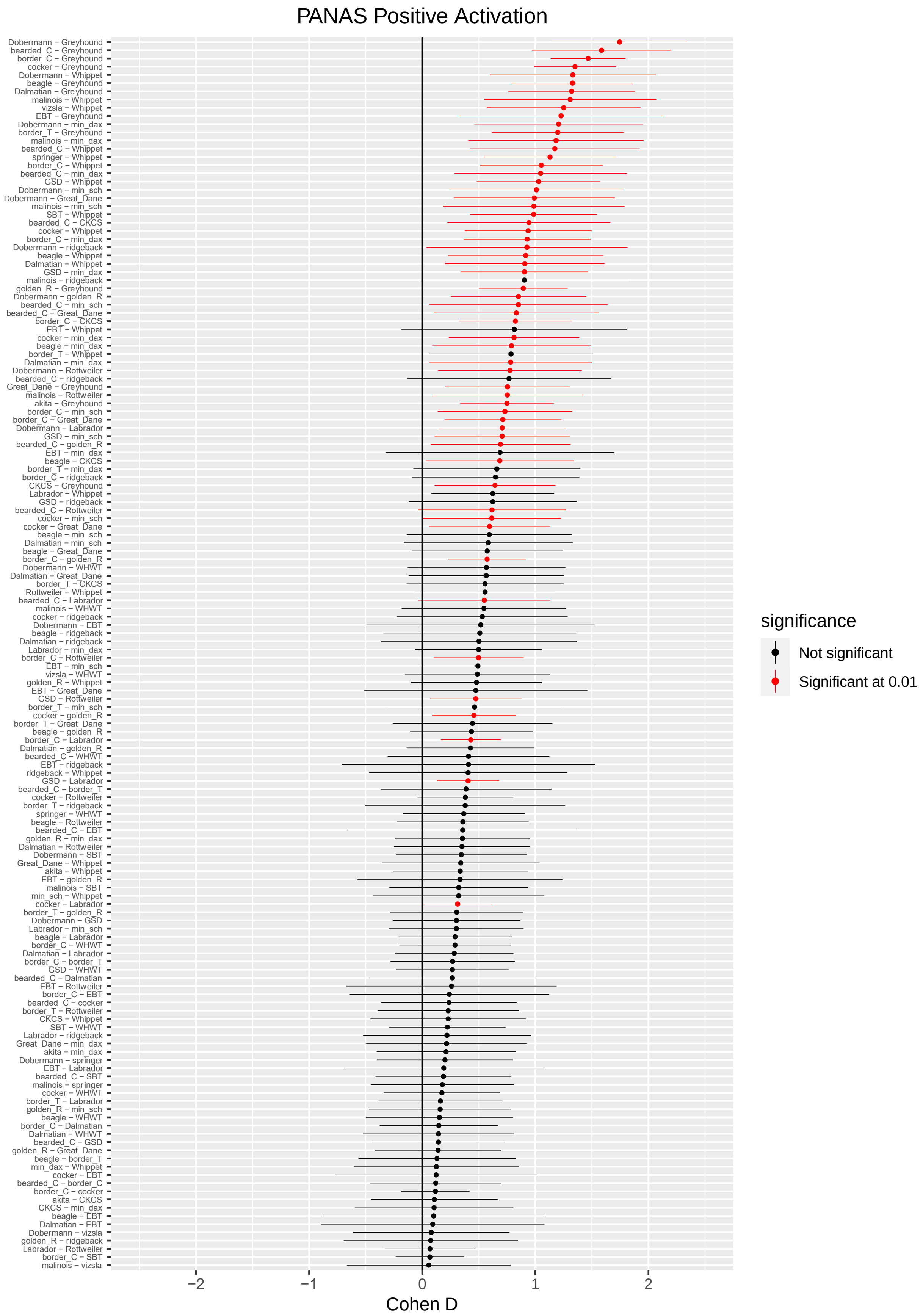

Supplementary Figure S17. Pairwise breed comparisons in PANAS Positive Activation. Cohen D point estimates are shown with 99% Confidence Intervals. Statistical significance shown refers to pairwise t-tests using the pooled standard deviation.

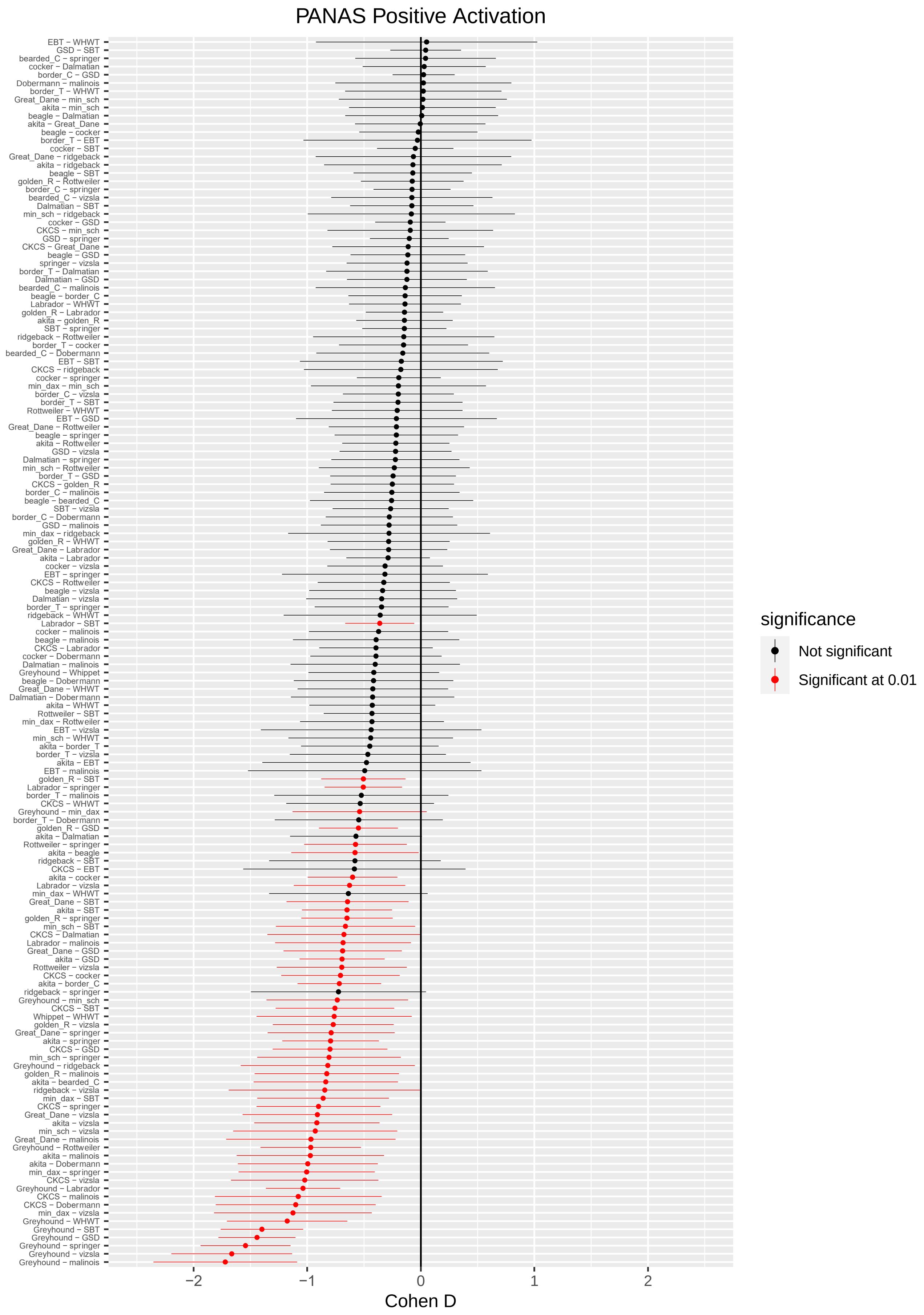

Supplementary Figure S17 continued.

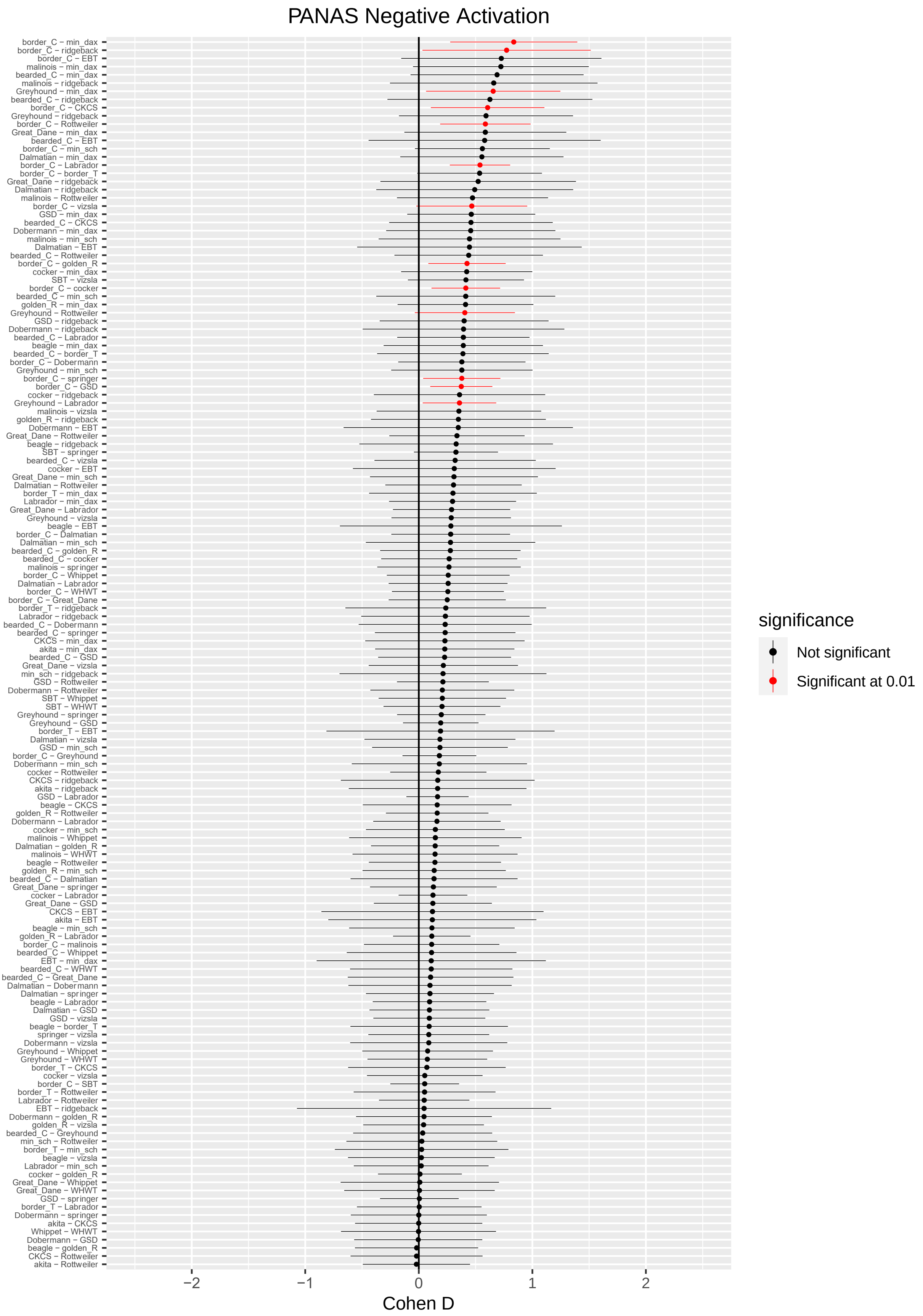

Supplementary Figure S18. Pairwise breed comparisons in PANAS Positive Activation. Cohen D point estimates are shown with 99% Confidence Intervals. Statistical significance shown refers to pairwise t-tests using the pooled standard deviation.

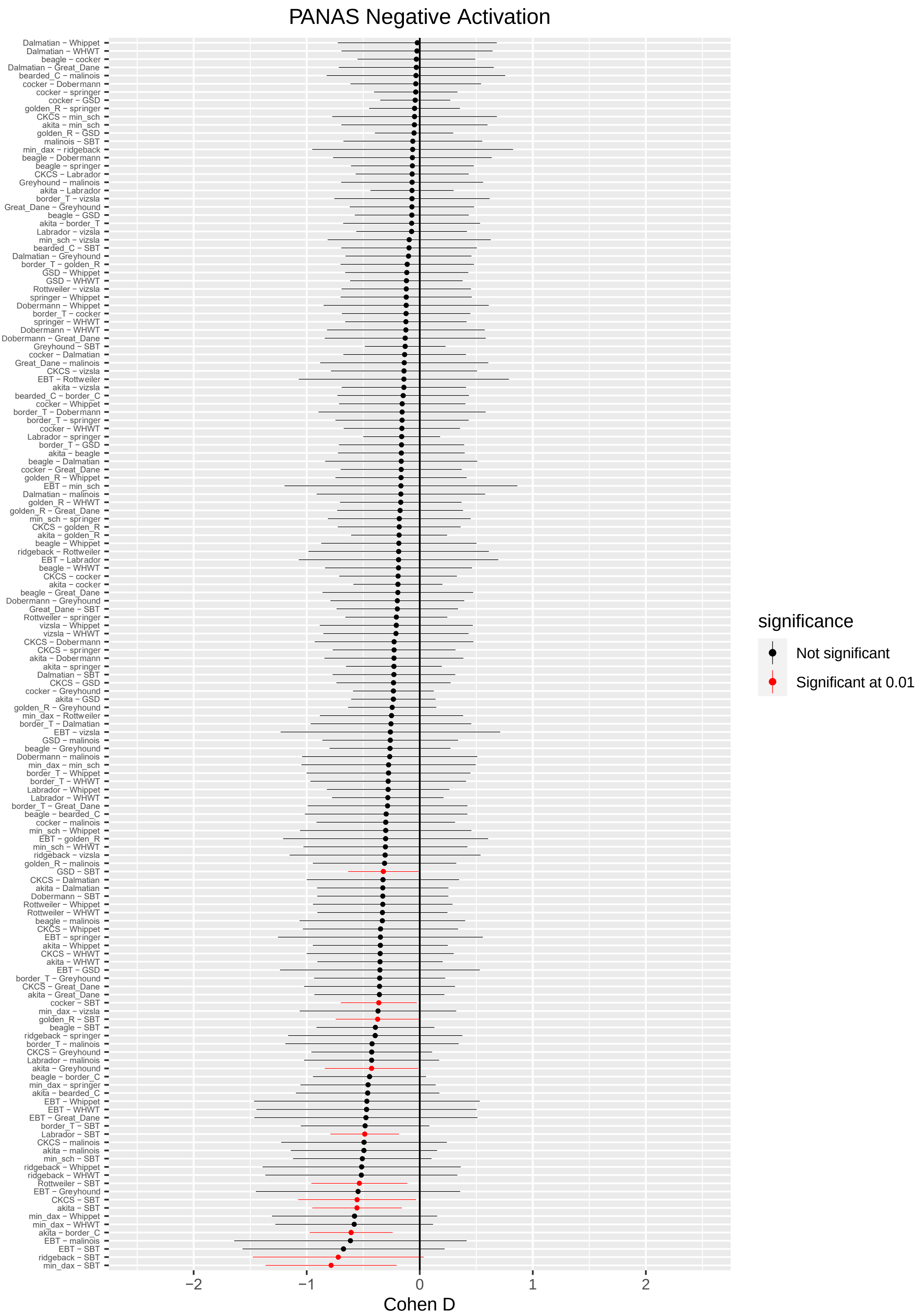

Supplementary Figure S18 continued.

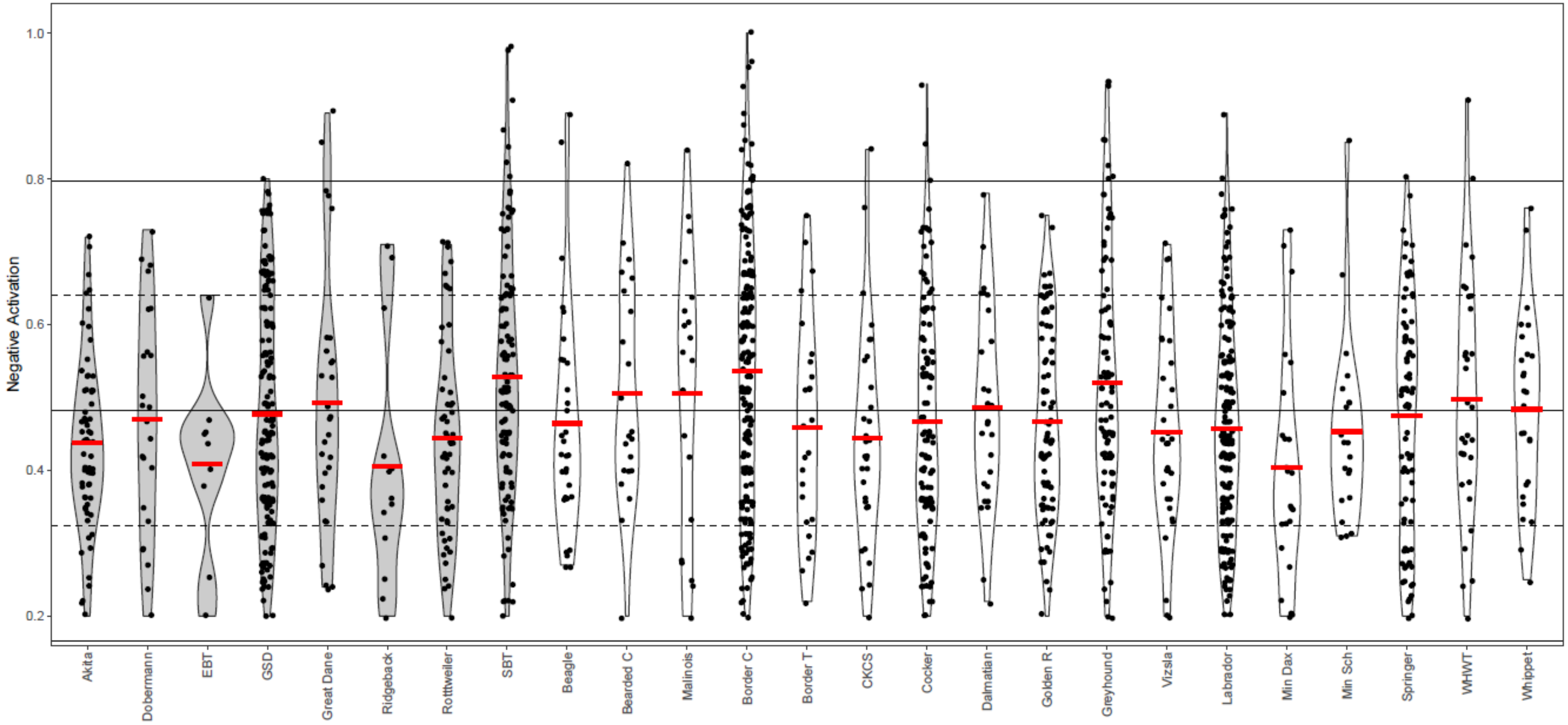

Supplementary Figure S19 A. Violin plot of PANAS Negative Activation in the 25 breeds used in the breed level analysis. Each black dot within the box is an individual's score. The solid horizontal red line through each box represents the respective breed mean. The solid horizontal line through the entire plot represents the total sample mean. The upper and lower dotted line represents +/-1 SD from the mean, and the upper solid line represents +/- 2 SD from the mean.

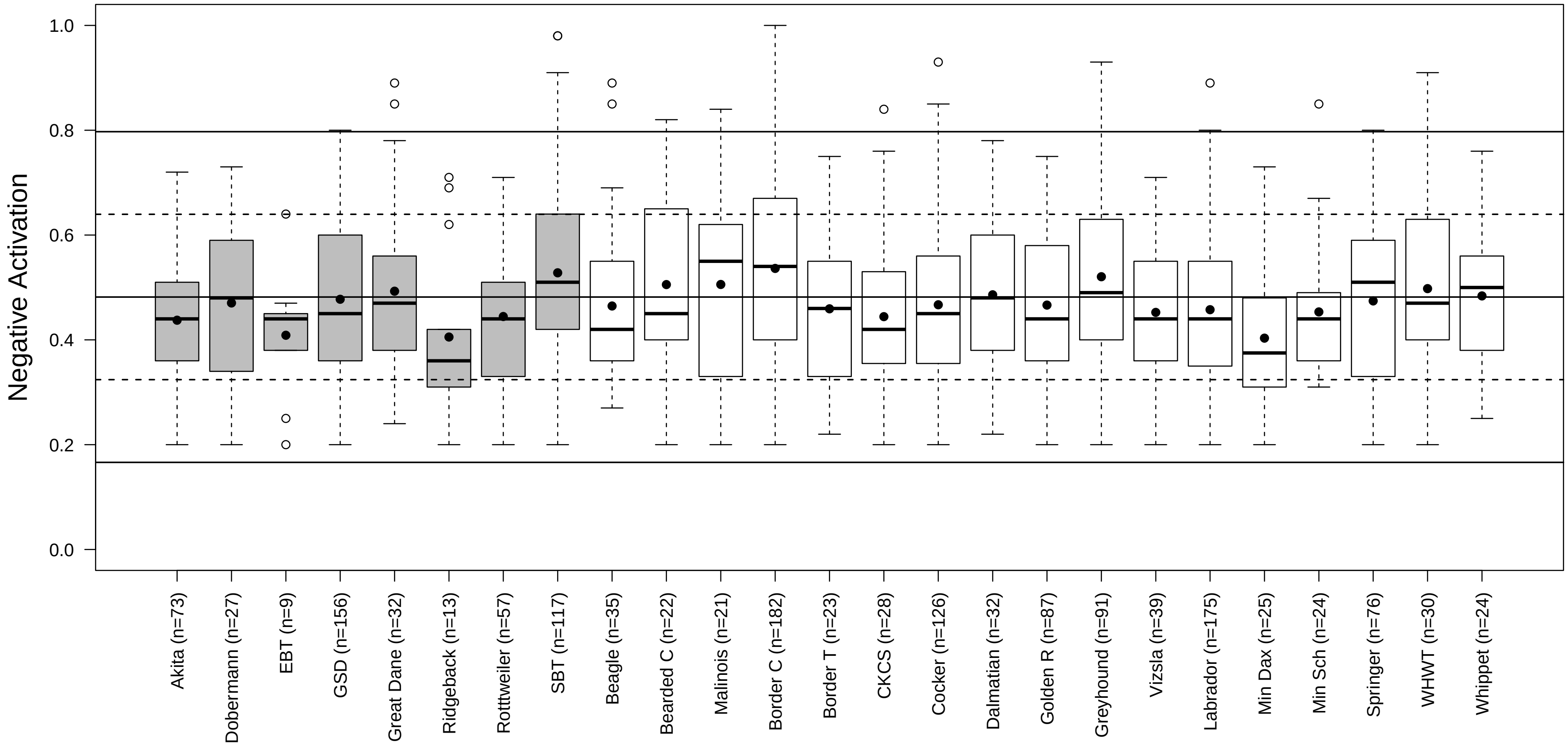

Supplementary Figure S19 B. Boxplot of PANAS Negative Activation in the 25 breeds used in the breed level analysis. Solid points on each box represents the respective breed means. Solid horizontal line through plot represents the whole sample mean, horizontal dotted lines represent ± 1SD, and the upper & lower horizontal solid lines represent ± 2SD.
